# Increasing sustainability and reproducibility of *in vitro* toxicology applications: serum-free cultivation of HepG2 cells

**DOI:** 10.3389/ftox.2024.1439031

**Published:** 2024-11-22

**Authors:** Luisa Marie Pfeifer, Janike Sensbach, Frederic Pipp, Daniela Werkmann, Philip Hewitt

**Affiliations:** ^1^ Early Investigative Toxicology, Merck Healthcare KGaA, Darmstadt, Germany; ^2^ Corporate Animal Affairs, Merck KGaA, Darmstadt, Germany; ^3^ Cell Design Lab, Molecular Biology, Merck KGaA, Darmstadt, Germany

**Keywords:** serum-free, *in vitro* toxicology, sustainability, animal welfare, HepG2

## Abstract

Fetal Bovine Serum (FBS) is an important ingredient in cell culture media and the current standard for most cells *in vitro*. However, the use of FBS is controversial for several reasons, including ethical concerns, political, and societal pressure, as well as scientific problems due to the undefined and variable nature of FBS. Nevertheless, scientists hesitate to change the paradigm without solid data de-risking the switch of their assays to alternatives. In this study, HepG2 cells, a human hepatoblastoma cell line commonly used to study drug hepatotoxicity, were adapted to serum-free conditions by using different commercially available media and FBS replacements. After transition to these new culture conditions, the success of adaptation was determined based on cell morphology and growth characteristics. Long-term culturing capacity for each medium was defined as the number of passages HepG2 cells could be cultured without any alterations in morphology or growth behavior. Two media (Advanced DMEM/F12 from ThermoFisher and TCM^®^ Serum Replacement from MP Biomedicals) showed a long-term cultivation capacity comparable to media containing FBS and were selected for further analysis. Both media can be characterized as serum-free, however still contain animal-derived components: bovine serum albumin (both media) and bovine transferrin (only TCM^®^ serum replacement). To assess the functionality of the cells cultivated in either of the two media, HepG2 cells were treated with reference compounds, specifically selected for their known hepatotoxicity characteristics in man. Different toxicological assays focusing on viability, mitochondrial toxicity, oxidative stress, and intracellular drug response were performed. Throughout the different assays, response to reference compounds was comparable, with a slightly higher sensitivity of serum-free cultivated HepG2 cells when assessing viability/cell death and a lower sensitivity towards oxidative stress. Taken together, the two selected media were shown to support growth, morphology, and function of serum-free cultivated HepG2 cells in the early preclinical safety space. Therefore, these results can serve as a starting point to further optimize culture conditions with the goal to remove any remaining animal-derived components.

## 1 Introduction

In pharmaceutical drug development, the use of *in vitro* cell, tissue and organ culture is an important tool to test drug candidates for efficacy, DMPL and toxicity. With increasing complexity of available cell culture models, great progress has been made to better approximate the human *in vivo* situation. These models have the potential of reducing the need for animal experiments in research and development (Ma et al., 2021; Wang et al., 2021). However, despite being considered a major alternative for animal experimentation, human *in vitro* cell culture systems often require animal components for supplementation (Jochems et al., 2002; van Zutphen et al., 2001; Weihe, 1985). Fetal bovine serum (FBS), Matrigel, and other animal derived products, such as growth factors, hormones, adhesion factors are commonly used to maintain and proliferate cells and tissues (Gospodarowicz and Moran, 1976; Hughes et al., 2010; Yao and Asayama, 2017).

FBS is an important ingredient in cell culture media and the current standard for media supplementation. It contains a highly complex mixture of components including proteins and peptides (adhesion molecules, carrier proteins, growth factors, hormones, etc.), vitamins, inorganic salts, amino acids, trace elements, carbohydrates, lipids and is able to support growth and survival of a variety of different cells and tissues (Gstraunthaler and Lindl, 2013; Maurer, 1986). In contrast to other sera, fetal calf serum is richer in growth factors while containing lower levels of γ-globulin, which is considered a cell growth inhibitor. For these reasons, FBS is the preferred cell culture supplement for *in vitro* experimentation (Gstraunthaler and Lindl, 2013).

However, using FBS in cell culture is highly questionable for several reasons. Ethical concerns arise due to the collection process for fetal calf blood. FBS, as a by-product of the beef industry, is collected when a cow sent for slaughter is discovered to be pregnant. Collection of fetal calf blood is done by cardiac puncture of at least 3-month-old calf fetuses, usually without any form of anesthesia (Jochems et al., 2002; van der Valk et al., 2004). A study conducted by Jochems et al. on the fetal bovine blood collection process drew the conclusion that fetuses are most likely exposed to pain and/or suffering, increasing the necessity to replace FBS in cell culture with an animal free alternative (Jochems et al., 2002).

Additionally, scientific problems arise due to the undefined nature and highly variable composition of FBS. Although FBS was introduced into cell culture already in the 1950s, it has never been fully characterized (Puck et al., 1958). Proteomic studies detected more than 3,000 proteins, with only a fraction of these being identified (Nakamura et al., 2021; Zheng et al., 2006). More studies have been conducted on human serum. Approximately 3,700 distinct proteins were found, of which 1800 proteins were identified (Anderson and Anderson, 2002; Anderson et al., 2004; Pieper et al., 2003). Furthermore, more than 4,000 metabolites are present in human serum (Psychogios et al., 2011).

Apart from the undefined nature, FBS is subject to seasonal and geographical lot-to-lot differences, which increases experimental variability (Baker, 2016; Zheng et al., 2006). Therefore, batch-to-batch differences are thought to play an important part in the well-known reproducibility problems when using serum and therefore force scientist to perform excessive batch-to-batch testing (Baker, 2016; Zheng et al., 2006). Additionally, FBS is also critical from a biosafety aspect, since FBS can be a source for contaminations such as *mycoplasma*, viruses, prion proteins or endotoxins (Merten, 2002; Nikfarjam and Farzaneh, 2012; Wessman and Levings, 1999).

Despite being able to support growth and maintain viability of different cells, factors present in FBS might interfere with the phenotypic stability of cells or interact with test substances, thus influencing experimental outcome (Khasawneh et al., 2019; Lau and Chang, 2014; Tang et al., 2017). As examples, Shahdadfar et al. demonstrated that FBS induced a more differentiated and less stable transcriptional profile in human bone marrow mesenchymal stem cells (hMSCs) compared to human serum while Bilgen et al. showed that FBS inhibited glycosaminoglycan and type II collagen production in fibroblast-like type-B synoviocytes (Bilgen et al., 2007; Shahdadfar et al., 2005). Despite all these described reasons, scientists hesitate to change the paradigm without solid data de-risking the switch of their assays to FBS-free culture media.

It has become clear that almost every cell type has its own requirements concerning medium supplements, since different cell types have different receptors involved in cell survival, growth and differentiation (van der Valk et al., 2010). Different alternatives to FBS have been suggested, including human platelet lysates (hPL), bovine ocular fluid, pituitary extracts, Earthworm heat inactivated coelomic fluid (HI-CF) or sericin (Bratko et al., 2002; Hammond et al., 1984; Johansson et al., 2003; Patil and Biradar, 2017; Rauch et al., 2011; Subbiahanadar Chelladurai et al., 2021; Terada et al., 2002). However, these alternatives are, like FBS, undefined and therefore scientific problems related to batch-to-batch variabilities are not eliminated. In order to tackle both, scientific and ethical problems in *in vitro* cell culture, approaches using serum-free media, protein-free media, animal-derived component free media (xeno-free media) and chemically defined media (See Table 1 for definition) are preferable. Implementation of such media is therefore recommended by the ECVAM Scientific Advisory Committee (ESAC) for current and new *in vitro* methods (Gstraunthaler et al., 2013; van der Valk et al., 2010). Notably, animal-derived component free or xeno-free media should be preferred, while serum-free media still containing animal-derived components should only be used if no suitable animal-derived component free or xeno-free alternative is available.

**TABLE 1:** Definition of cell culture media adapted from van der Valk et al. (2010).

Media type	Definition
Serum-free medium	Does not contain serum but may contain discrete proteins or bulk protein fractions (e.g., animal or human tissue or plant extracts). Therefore, the media is regarded as undefined
Protein-free medium	Does not contain high molecular weight proteins or protein fractions, but can contain peptide fractions (protein hydrolysates), therefore the medium is not chemically defined
Xeno-free medium	Does not incorporate compounds from foreign species. Medium intended for use on human cells only contains human components
Animal-derived component free media	Does not contain components of human or animal origin
Chemically defined media	Does not contain components of unknown composition like hydrolysates. May contain highly purified growth factors or hormones, either recombinant or of human, animal, or plant origin

Since different cells and cell lines rely on different factors for survival or proliferation, the development of a chemically defined medium is not always straight forward and needs to be properly assessed. To simplify the development process, van der Valk et al. proposed a modular approach starting off with a 1:1 mixture of DMEM and Ham-F12 supplemented with insulin, transferrin and sodium selenite (van der Valk et al., 2010). For adherent cells and cell lines, adhesion factors like fibronectin, vitronectin, collagen or laminin are typically used for pre-coating of the culture vessels. Preferably, these adhesion factors should be either human or recombinant in order to avoid animal components in cell culture. Subsequently, specific hormones and growth factors may be supplemented. In the last step, lipids, antioxidants and/or vitamins are added to the medium. With increasing complexity of the serum-free media composition, specificity is increased as well (van der Valk et al., 2010). Therefore, development of cell or cell type specific media have been often preferred over a universal chemical defined medium, especially when considering production costs for growth factors and other proteins (Karnieli et al., 2017; van der Valk et al., 2010; Yao and Asayama, 2017).

**FIGURE 1:**
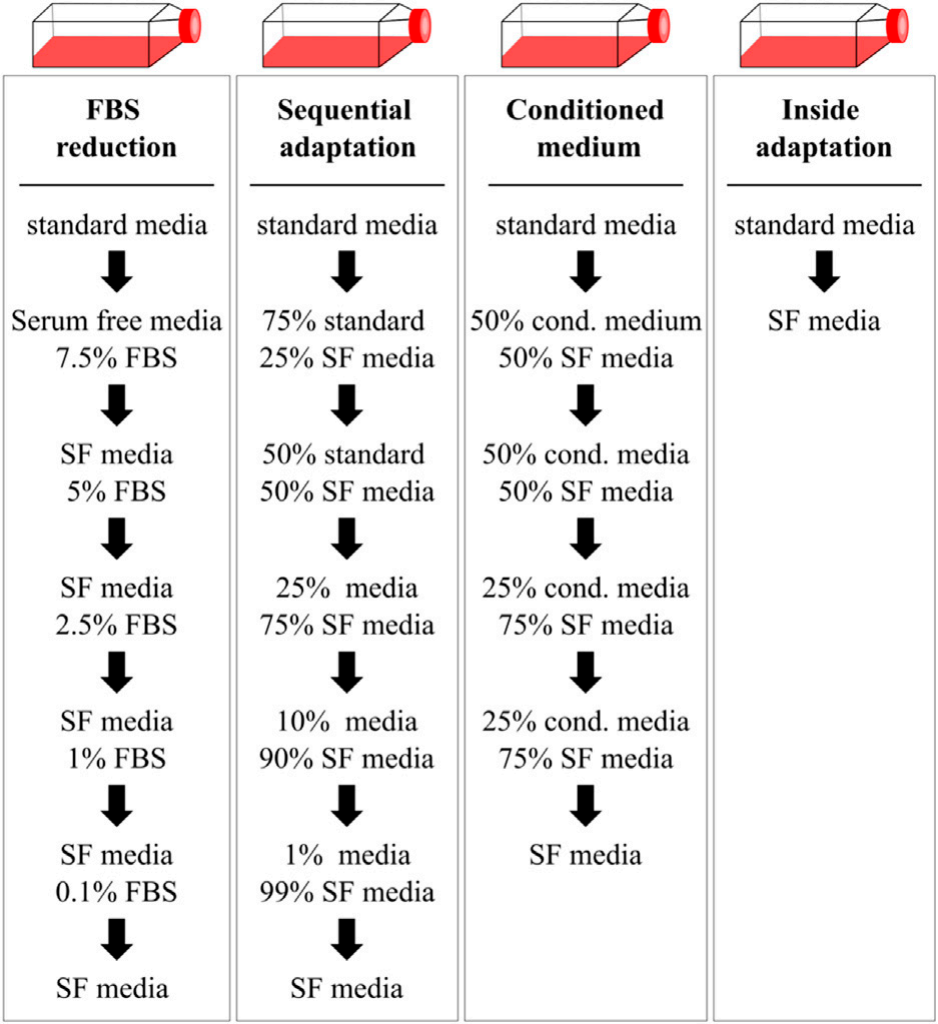
Gradual and immediate adaptation mechanisms as described by Van-der-Valk et al. used to transfer HepG2 cells in serum-free conditions. Cells were transferred to serum-free media (SF media) either by gradually reducing the FBS content (FBS reduction), mixing of the serum-free medium with the standard medium containing FBS (sequential adaptation) or by supplementing the serum-free medium with conditioned medium of the previous passage. Alternatively, cells were immediately transferred to serum-free conditions during the splitting process (inside adaptation). Cells were transferred to the next condition if viability was above 90%.

Several different FBS free media have been developed, both cell type specific and universal, with a steady growth with progressing scientific progress (Karnieli et al., 2017). Media formulations from literature are collected within the Fetal Calf Serum free Database (RRID:SCR_018769; https://fcs-free.org), while formulations of commercial media are usually proprietary. Finding a suitable media to maintain the desired cells and tissues and support the experimental goals can be challenging, especially when an immediate transfer of cells to serum free conditions is not possible. In this case, cells need to be adapted carefully in a time-consuming process. Different protocols to do this have been proposed to adapt cells to serum-free conditions (see Figure 1).

Dependent on the cell type or cell line, serum-free culture may either be already commonly performed and described frequently in the literature, while for other cell types, fewer resources may be available. Under the scope of liver models for toxicity testing, serum-free culture is routinely used to cultivate primary human hepatocytes, in both 2D or 3D applications (Bell et al., 2016; Klaas et al., 2021; Lübberstedt et al., 2015; Runge et al., 2000). Additionally, serum-free culture was demonstrated for HepaRG cells, an immortalized hepatic cell line, as well as different liver cancer derived cell lines, e.g., Huh7, SK-Hepp-1 and HKB-11 cells (Abe et al., 2007; Akazawa et al., 2011; Biaggio et al., 2015; Klein et al., 2014). However, these serum-free cultivated liver cancer cells have not yet been tested for specific toxicity applications.

The aim of this study was to adapt HepG2 cells to serum-free conditions using different commercially available media and subsequently test serum-free cultivated cells in comparison to cells cultivated in serum containing medium in standard assays which are sued for assessing cytotoxicity and hepatotoxicity.

HepG2 cells are a popular highly proliferative hepatic cell line with epithelial-like morphology that grows as monolayers and form characteristic cell clusters or islands (Vinken and Rogiers, 2015). According to ATCC, this cell line was isolated from a hepatocellular carcinoma (HCC) of a 15-year-old, White, male youth with liver cancer, however more recent findings suggest that the HepG2 cell line is instead a hepatoblastoma (HB) cell line (Arzumanian et al., 2021; *Hep G2 [HB-8065] | ATCC*). This cell line can be cultured in different media (Dulbecco’s Modified Eagle Medium, Dulbecco’s Modified Eagle Medium/Nutrient Mixture F-12, Eagle’s Minimum Essential medium, or Roswell Park Memorial Institute 1,640 medium) supplemented with 10% FBS, and, dependent on the used media, L-glutamine and/or sodium pyruvate (*Hep G2 [HB-8065] | ATCC*; Luckert et al., 2016; Naito et al., 2018; Vinken and Rogiers, 2015). So far, serum-free culture of HepG2 cells has been shown mainly for short term applications, especially for 3D spheroids or multi-organ models or very short-term applications under serum-starvation (Gunn et al., 2017; Oleaga et al., 2016; Sefried et al., 2018; Sun et al., 2024; Wrzesinski et al., 2013). Since only a limited number of animal-derived component free or xeno-free media was deemed suitable, serum-free media were included as well, if the percentage of animal-derived components within the media was low. Results on serum-free medium should then be used to further optimize culture conditions towards animal-derived component free culture in future experiments.

Cells adapted to serum-free conditions should maintain morphological characteristics and display similar high proliferation rates–both parameters were being used in this work. With a wide range of applications, the HepG2 cell line is most often used in early drug development, to test for cytotoxicity and hepatotoxicity (Vinken and Rogiers, 2015). Therefore, the key aspect of this study was on the comparability of serum-free cultured HepG2 in common toxicological assays. To compare serum-free HepG2 cells with cells cultured in FBS containing media, cells were treated with a broad range of reference compounds, selected from the IMI project “mechanism-based integrated prediction of drug-induced liver injury in man” (MIP-DILI), internal reference compounds or negative control compounds (Dragovic et al., 2016). These compounds exhibit different mechanisms of DILI including: mitochondrial impairment, reactive metabolites, lysosomal impairment, BSEP inhibition and/or innate/adaptive immune activation.

## 2 Experimental procedures

### 2.1 Materials

All reference compounds were purchased from Sigma-Aldrich (St.Louis, MO) unless otherwise noted. Reference compounds are depicted in Table 2.

**TABLE 2:** Reference compounds used in toxicological assays.

	Compound	Classification	Clinical availability	Human DILI	References
MIP-DILI Compounds	Acetaminophen	COX inhibitor	Yes	Most DILI concern (1,2)	McClain et al. (1999)
	Amiodarone	Class III antiarrhythmic medication	Black-box warning	Most DILI concern (1,3)	Heath et al. (1985), Pirovino et al. (1988)
	Bosentan	ETAR/ETBR inhibitor	Yes	Most DILI concern (4)	Fattinger et al. (2001)
	Diclofenac	COX inhibitor	Yes	Most DILI concern (1,2,4)	Gómez-Lechón et al. (2003), Tang (2005)
	Fialuridine	Nucleoside analogue	No	Most DILI concern (1)	Kleiner et al. (1997)
	Flucloxacillin	Antibiotic	Yes	Most DILI concern (5)	Monshi et al. (2013)
	Nefazodone	5-HT2 receptors inhibitor	Withdrawn	Most DILI concern (2,4)	Kalgutkar et al. (2005), Kostrubsky et al. (2006)
	Perhexiline	CPT1/2 inhibitor	Yes	Most DILI concern (3,4)	Ashrafian et al. (2007)
	Tolcapone	COMT inhibitor	Black-box warning	Most DILI concern (2,4)	Haasio et al. (2002), Longo et al. (2016)
	Troglitazone	PPARγ agonist	Withdrawn	Most DILI concern (1,2,3,4)	Smith (2003)
	Ximelagatran	Anticoagulant	Withdrawn	Most DILI concern (5)	Kindmark et al. (2007)
MIP-DILI Negative Controls	Buspirone	5HT(1A) receptor agonist	Yes	Ambiguous DILI concern	Dykens et al. (2008)
	Entacapone	COMT inhibitor	Yes	Less DILI concern	Longo et al. (2016)
	Metformin	AMP-activated protein kinase activator	Yes	Less DILI concern	Shurrab and Arafa (2020)
	Pioglitazone	PPARγ agonist	Yes	Less DILI concern	Rajagopalan et al. (2005)
Internal controls	Tamoxifen	Estrogen receptor antagonist	Yes	Most DILI concern	Gao et al. (2016), Lin et al. (2014)
	Clotrimazole	Antifungal	Yes	Less DILI concern	Yong et al. (2007)
	Buthionine sulfoximine (BSO)	γ-glutamylcysteine synthetase inhibitor	No	Unknown	O'Dwyer et al. (2016)
	Thapsigargin	Non-competitive SERCA inhibitor	No	Unknown	Thastrup et al. (1990)
	Paclitaxel	Tubulin depolymerization inhibitor	Yes	Less DILI concern	Marupudi et al. (2007)
	Propranolol	Beta-adrenergic receptor blocker	Yes	Ambiguous DILI concern	McKee et al. (2013), Routledge and Shand (1979)
	Cyclosporin A	Calcineurin inhibitor	Yes	Most DILI concern	Kassianides et al. (1990), Korolczuk et al. (2016)

*1* mitochondrial, *2* reactive metabolites, *3* lysosomal impairment, *4* BSEP inhibition, *5* immune-mediated (Dragovic et al., 2016).

### 2.2 Cell culturing (standard procedure with FBS)

HepG2 (HB-8065, ATCC) were cultured in Dulbecco’s Modified Eagle Medium/Nutrient Mixture F-12 (DMEM/F12 with HEPES, 31330) supplemented with 10% fetal bovine serum and 1 mM sodium pyruvate (all Gibco™) at 37°C and 5% CO_2_. Cells were split twice per week using TrypLE™ (Gibco™, 12604013) for detachment. Subcultivation ratios ranged between 1:3 and 1:5, corresponding to ∼8–10 × 10^4^ cells/cm^2^. Cells were used for up to 30 passages before disposal.

### 2.3 Comparison of adaptation procedures

For a comparison of adaptation processes, HepG2 cells were adapted to two different serum-free media, serum-free medium 1 (SF-M1; Advanced DMEM/F12) or serum-free medium 3 (SF-M3), by FBS reduction, sequential adaptation, by using conditioned medium or inside adaptation as described in Figure 1 (van der Valk et al., 2010). Media characterization can be found in Supplementary Table S1. Subcultivation was performed when a confluency of ∼70–80% was reached. Cell media was changed in case of slower growth every 2–3 days. Cells were maintained in one condition for at least two passages before lowering the FBS concentration again. Viability and cell density were taken as parameters to decide whether to maintain cells in one particular condition or continue the adaptation process. Vitronectin coating (using VTN-N, A14700, ThermoFisher, 0.5 μg/cm^2^) was applied if a reduction in cellular adherence was observed.

### 2.4 Adaption of HepG2 cells to serum-free conditions

Cells were adapted to serum-free conditions using the inside adaptation strategy proposed by van der Valk et al. (2010) (Figure 1). Different products were tested for cultivation of HepG2 (Supplementary Table S1). Serum-free media (SF media) were supplemented with HEPES, sodium pyruvate (1 mM) or GlutaMax (2 mM) if not present in the medium already.

### 2.5 Growth curve and doubling time determination

Growth of cells cultivated in standard medium containing FBS, Advanced DMEM/F12 or TCM^®^ Serum Replacement was assessed using the Incucyte^®^ S3 Live-Cell Analysis Instrument from Sartorius. HepG2 were seeded in 6-well plates at 8 × 10^4^ cells/cm^2^. For serum-free conditions, plates were coated with vitronectin (0.5 μg/cm^2^). Medium was changed after 3 days for all conditions. Confluency was determined every 8 h using 4 pictures per well. Growth curves were modeled in GraphPad Prism from confluency values using a logistic growth model. Doubling times were calculated on the exponential (log) phase using an exponential (Malthusian) growth model.

### 2.6 Immunofluorescent staining

HepG2 cells cultivated in standard medium containing FBS, Advanced DMEM/F12 or TCM^®^ Serum Replacement were stained for nuclei (DAPI, D9542-10 MG, Millipore Sigma), actin (A12379, Invitrogen), albumin (sc50535, Santa Cruz Biotechnology) and alpha-1-antitrypsin (PA5-16661, Invitrogen). Cells were cultured in 96-well plates for 3 days. For staining, media was aspirated, and cells were fixed with 4% formaldehyde for 30 min. Afterwards, cells were washed two times with Wash buffer (PBS +/+, 0.2% Triton X-100, 0.04% Tween-20) and incubated for 5 min with permeabilization buffer (PBS +/+, 0.1% Triton X-100). Before staining, blocking buffer (PBS +/+, 5% BSA, 0.2% Triton X-100, 0.04% Tween-20) was added to the cells for 30 min. Buffer was aspirated and cells were incubated with primary antibodies over night at 4°C.

The next day, the primary antibodies were aspirated, the cells were washed twice with wash buffer before the secondary staining antibody (Alexa Fluor 680 goat-anti-rabbit, A21076, Life technologies) was added. Cells were incubated for 1 h at RT, washed twice and subsequently imaged using the high content imager CellInsight CX7 from Thermo Fisher.

Notably, antibodies and BSA are animal-derived components and should therefore be avoided.

### 2.7 Assessment of urea and albumin secretion

Urea and human albumin secretion was measured in cell culture supernatants of cells cultivated in standard medium containing FBS, Advanced DMEM/F12 or TCM^®^ Serum Replacement. Cells were grown over 96 h and samples were taken after 6 h, 48 h and 96 h. Cells were counted after sample collection. Human albumin concentration was measured using the human albumin ELISA kit (ab179887) from Abcam according to manufacturer’s instructions. Urea concentrations were determined using the urea assay kit (MAK006) from Sigma-Aldrich. Standard curves for both assays were generated using a sigmoidal, four parameter curve fit (4 PL).

### 2.8 CellTiter-Glo^®^ assay

Cell viability in response to compound treatment was accessed by measuring ATP using the CellTiter-Glo^®^ Luminescent Cell Viability Assay from Promega (G7571) according to manufacturer´s instructions. HepG2 cells cultivated in standard medium containing FBS, Advanced DMEM/F12 or TCM^®^ Serum Replacement were seeded in 384-well plates, incubated for 24 h, and subsequently treated with the MIP-DILI reference compounds, negative controls, or internal controls in a three-fold dilution series performed in triplicates using the Tecan 300e Dispenser (Dragovic et al., 2016). Treatment was performed for 24 h.

CellTiter-Glo^®^ substrate was dissolved in CellTiter-Glo^®^ buffer and equilibrated at room temperature prior to use. Plates were incubated at room temperature for 30 min before adding the CellTiter-Glo^®^ reagent. Subsequently, plates were shaken for 2 min protected from light and incubated for 10 min in the dark. Luminescence was measured using the Varioskan Plate Reader from Thermo Fisher. IC_50_ values were obtained from dose response curves in GraphPad Prism using a log(inhibitor) vs. response model (variable slope, four parameters, bottom constraint = 0).

### 2.9 Alamar blue assay

Metabolic activity of cells in response to compound treatment was accessed using the Alamar Blue Assay. HepG2 cells cultivated in standard medium containing FBS, Advanced DMEM/F12 or TCM^®^ Serum Replacement were seeded in 384-well plates, incubated for 24 h, and subsequently treated with the MIP-DILI reference compounds or negative controls in a three-fold dilution series performed in triplicates using the Tecan 300e Dispenser (Dragovic et al., 2016). Treatment was performed for 24 h.

Resazurin (Sigma-Aldrich, R7017-5G) was dissolved in PBS solution at 4.5 mM and added to as 10% of total volume per well. Plates were incubated for 2 h. After incubation, the supernatant was transferred into black, clear bottom 384-well plates and protected from light until measurement. Fluorescence was measured at λ excitation: 571 nm; λ emission: 585 nm using the Varioskan Plate Reader from Thermo Fisher. IC_50_ values were obtained from dose response curves in GraphPad Prism using a log(inhibitor) vs. response model (variable slope, four parameters, bottom constraint = 0).

### 2.10 Glutathione (GSH) assay

To assess oxidative stress in cells in response to compound treatment, the GSH-Glo™ Glutathione Assay from Promega was used according to manufacturer’s instructions (V6912). HepG2 cells cultivated in standard medium containing FBS, Advanced DMEM/F12 or TCM^®^ Serum Replacement were seeded in 384-well plates, incubated for 24 h, and subsequently treated with the MIP-DILI reference compounds, negative controls, or internal controls in a three-fold dilution series performed in triplicates using the Tecan 300e Dispenser (Dragovic et al., 2016). Treatment was performed for 24 h.

Assay reagents and plates were first equilibrated to room temperature. GSH-Glo™ Reagent 1x was obtained by diluting Luciferin-NT substrate and Glutathione S-Transferase 1:100 in GSH-Glo™ Reaction Buffer. Luciferin Detection reagent was dissolved in Reconstitution Buffer with Esterase.

The medium was aspirated, GSH-Glo™ Reagent 1x was added to the cells and plates were shaken for 2 min and subsequently incubated for 30 min at room temperature protected from light. Afterwards, reconstituted Luciferin Detection Reagent was added, and cells were again shaken for 2 min before incubation for 15 min at room temperature in the dark. Luminescence was measured using the Varioskan Plate Reader from Thermo Fisher.

### 2.11 Mitochondrial toxicity assay

To assess mitochondrial toxicity, the Glucose/Galactose Assay was used. HepG2 cells cultivated in standard medium containing FBS, Advanced DMEM/F12 or TCM^®^ Serum Replacement were seeded in 384-well plates and incubated for 48 h. Cells were washed once, and subsequently switched to DMEM (ThermoFisher, 11966025) supplemented with sodium pyruvate, HEPES and either 25 mM D-glucose or 10 mM D-galactose without FBS to avoid residual glucose. Cells were pre-incubated for 4 h before treatment with the MIP-DILI reference compounds, negative controls or internal controls (Dragovic et al., 2016). Treatment was performed in a three-fold dilution series in triplicates using the Tecan 300e Dispenser. Cells were treated for 2 h, and the read-out was performed by measuring cell viability using the CellTiter-Glo^®^ reagent as described previously. IC_50_ values were obtained from dose response curves in GraphPad Prism using a log(inhibitor) vs. response model (variable slope, four parameters, bottom constraint = 0). Mitochondrial toxicity was assessed by calculating the IC_50_Glc/IC_50_Gal ratio and the threshold for risk for mitochondrial toxicity was set at >2.

### 2.12 Measurement of intracellular drug response markers (cellular fingerprint assay)

To measure intracellular drug response markers, HepG2 cells cultivated in standard medium containing FBS, Advanced DMEM/F12 or TCM^®^ Serum Replacement were seeded in 384-well plates and incubated for 24 h. After incubation, cells were treated with selected MIP-DILI compounds or negative controls in a three-fold dilution series performed in triplicates using the Tecan 300e Dispenser (Dragovic et al., 2016). Additionally, cells were treated with assay specific control substances (see Supplementary Table S2). Treatment was performed for 24 h. After treatment, media was carefully aspirated und cells were lysed by adding 40 µL lysis buffer (Supplementary Table S3) to each well. The plates were incubated for 30 min at 4°C and either stored at −80°C or used immediately.

Intracellular drug response markers were assessed using the Luminex technology. Color-coded beads from NMI specific for one analyte each were used to qualify and quantify p-serin 10 of Histon H3 (cell division marker), LC3B (autophagy marker), PARP (cell death/apoptosis marker), HSP70 (stress response marker), p-serin 2/5 of RNA-Polymerase II (protein transcription marker), p-serin 422 of EIF4B (protein synthesis marker) and HIF1-alpha (oxidative stress marker).

For hybridization, 25 μL cell lysate of each sample was transferred to a fresh 384-well plate. 25 μL prepared bead mix and 25 µL assay buffer were added and the plate was sealed. Plates were incubated for 16–20 h at 4°C and 600 rpm. The TS Pro thermal plate shaker from CellMedia was used for all incubation steps.

After incubation the plates were washed and successively incubated with 15 µL detection antibody (1 h, RT, 650 rpm) and 15 µL streptavidin, R-phycoerythrin conjugate (SAPE; 45 min, RT, 650 rpm). After each step, the plates were washed three times with wash buffer. Before measurement, 90 µL assay buffer was added to each well and the plates were incubated for 10 min at RT and 700 rpm. Measurement was performed using the Luminex™ FlexMAP 3D from ThermoFisher.

### 2.13 Comparison of freezing media

Different freezing media were tested for their potential to store serum-free cultivated HepG2 as depicted in Table 3. HepG2 cells cultivated in standard medium containing FBS, Advanced DMEM/F12 or TCM^®^ Serum Replacement were detached using TrypLE™ and resuspended in media. After centrifugation, medium was aspirated, and cells were resuspended in freezing media. Commercial media were used according to manufacturer’s instructions. For conditioned medium, 45% fresh medium, 45% conditioned medium and 10% DMSO was used. Cells were transferred to cryogenic storage vials and frozen using NALGENE™ Cryo 1°C Freezing containers (ThermoFisher, 5100–0001).

**TABLE 3:** Freezing media tested for storage of serum-free cultivated HepG2.

Product	Supplier (Catalogue number)	Classification
CryoStor^®^ CS10	StemCell (100–1,061)	Animal-component free, protein-free
CryoPan I	PAN Biotech (P07-92500)	Serum-free
Cryo-SFM	PromoCell (C-2991)	Animal-component free, protein-free
Conditioned medium		Serum-free

### 2.14 Statistical analysis

All results are presented as mean ± standard deviation if not stated otherwise. Analysis was performed using the GraphPad prism software. All statistical test performed, as well as the number of replicates are summarized in Supplementary Table S4. Results were considered significant if adjusted *p* < 0.05.

### 2.15 Ethical statement

The materials used for this research do not meet the standard of being completely free from animal derived components.

We are committed to continuous improvement in our sourcing and manufacturing processes to comply with our high ethical standards and growing societal expectations.

## 3 Results

### 3.1 Comparison of adaptation protocols for transition of HepG2 to serum-free conditions

**FIGURE 2:**
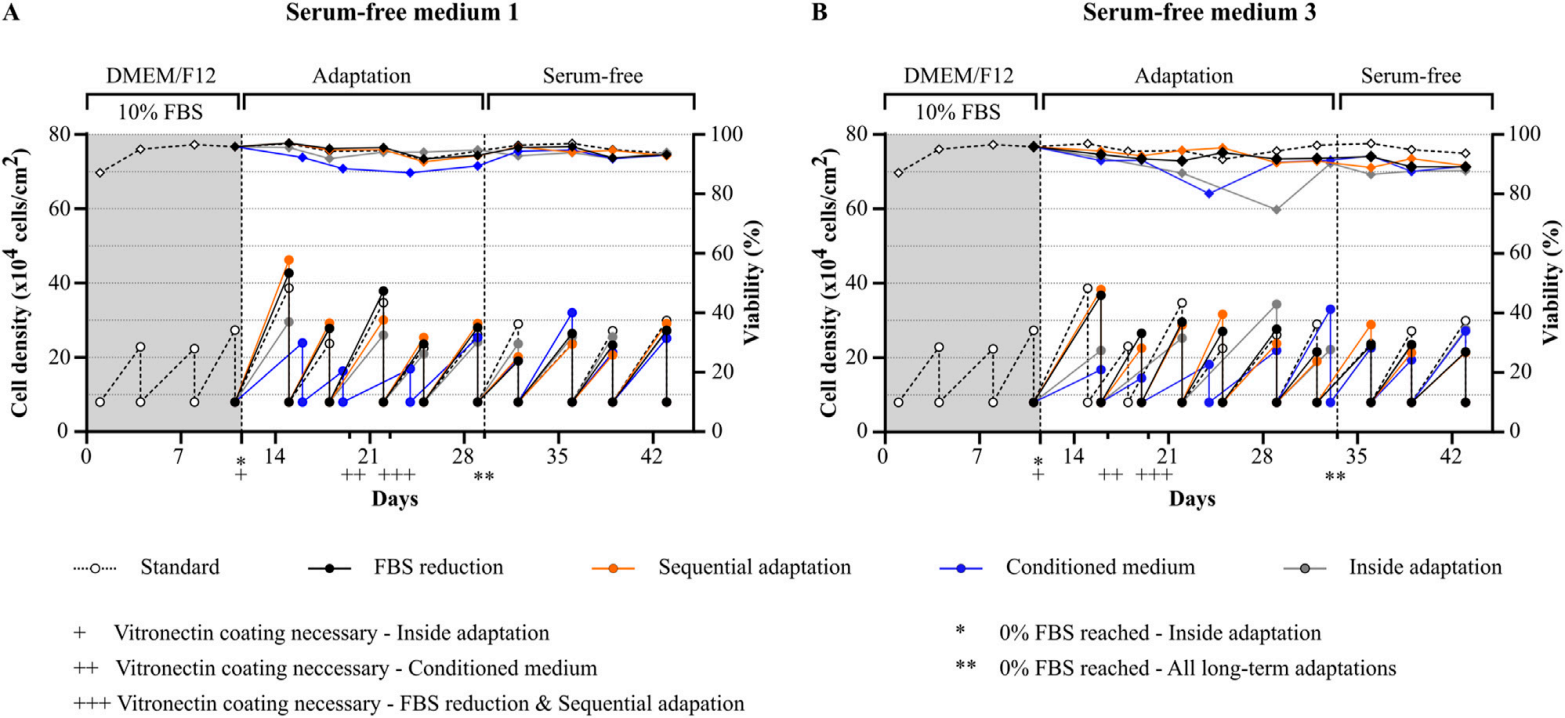
Comparison of adaptation protocols for transitioning of HepG2 cells to serum-free conditions. Viability (rhombuses) and viable cell density (circles) of cells during and after the adaptation process using different protocols (FBS reduction, sequential adaptation, conditioned medium or inside adaptation). Two randomly selected serum-free media were used: **(A)** serum-free medium 1 and **(B)** serum-free medium 3. Vitronectin coating was required for successful serum-free culture and was mandatory for all protocols with different starting points as indicated (+: inside adaptation, ++ conditioned medium, +++ FBS reduction and sequential adaptation). FBS-free conditions for the long-term adaptation protocols were reached at day 29 for serum-free medium 1 and Day 32 for serum-free medium 3 as indicated. Cells were splitted to the same cell density during each splitting process (8 × 10^4^ cells/cm^2^).

The four different adaptation protocols described by Van-der-Valk et al. (FBS reduction, sequential adaptation, conditioned medium and inside adaptation, see Figure 1) were compared for the transitioning of HepG2 to serum-free conditions (van der Valk et al., 2010). Ten different commercially available products (either serum-free media or FBS replacements) were purchased (Supplementary Table S1). Of these products, two media (serum-free medium 1 and serum-free medium 3) were randomly selected and cells were either gradually or immediately transitioned to serum-free conditions (Figure 2). For gradual adaptation of the cells, serum was reduced during passaging, whenever the viability of the culture was higher than 90%. In case that the cells displayed a viability lower than 90%, cells were kept in the same condition for another passage.

In general, the two media showed a different performance. While it was possible to maintain the cells after adaptation in serum-free media 1 with similar growth rates and a high viability comparable to cells cultured under FBS containing conditions, cells cultured in serum-free medium 3 were less viable throughout the whole experiment.

For cells adapted to serum-free medium 1 (Figure 2A), positive results were obtained with all four protocols. No differences were observed between FBS reduction and sequential adaptation, and viability and growth rates remained comparable to the control condition during the whole experiment. The use of conditioned medium during the adaptation process led to a significant reduction in both viability and growth, which normalized again after reaching 0% FBS. Finally, cells transitioned to FBS free medium using the inside adaptation protocol maintained a high viability but showed slightly reduced growth rates in the first three serum-free passages (day 11 till day 22) which subsequently normalized again.

For cells adapted to serum-free medium 3 (Figure 2B), the best results were obtained by using either the FBS reduction or sequential adaptation approach. Nevertheless, a reduction in viability and growth was observed during the adaptation process which did not normalize after reaching 0% FBS. For the conditioned medium approach, and the inside adaptation protocol, viability and growth rates dropped significantly. In both cases, growth and viability improved after several passages, however, as observed for FBS reduction and sequential adaptation, cells were not as viable as cells cultured in the FBS-containing condition.

Taken together, we observed that gradual adaptation using either FBS reduction or sequential adaptation is a gentler approach which allowed cells to maintain high viability and comparable cell growth at the expense of a prolonged time frame. In contrast, inside adaptation allowed for fast transitioning of cells to serum-free conditions at the expense of cell growth and viability, especially if the media formulation is not specifically designed for HepG2 cells. However, as inside adaptation allows for a fast differentiation between suitable and less suitable serum-free media and cells transitioned to serum-free medium 1 showed comparable growth and viability after already 3 passages, follow-up experiments were conducted using this approach.

### 3.2 Serum-free medium–front-runner selection

Cells were adapted to the different serum-free media or media supplemented with FBS replacements using the inside adaptation approach (Supplementary Table S1). After transition to the new culture conditions, the success of adaptation was determined based on cell morphology and growth characteristics. In addition, the long-term culturing capacity for each medium was defined as the number of passages HepG2 could be cultured without any alterations in these two parameters. If cells displayed significant changes in growth behavior or morphology, the culture was terminated. Since the aim of this experiment was to find the most suitable serum-free growth media, the possibility of cells to recover after multiple passages was not assessed.

**FIGURE 3:**
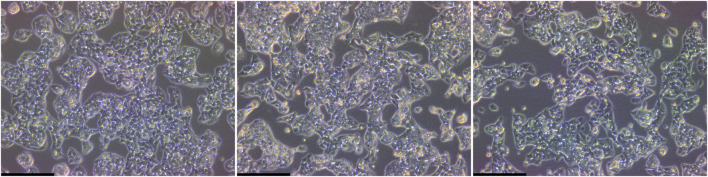
Analysis of morphology of serum-free cultivated HepG2. Microscopic images of cells cultivated under serum containing conditions (left), in Advanced DMEM/F12 (ThermoFisher, middle) or DMEM/F12 supplemented with TCM^®^ defined serum replacement (MP Biomedicals, right). Vitronectin coating was used for serum-free conditions to facilitate attachment. Scalebar: 250 µm.

**FIGURE 4:**
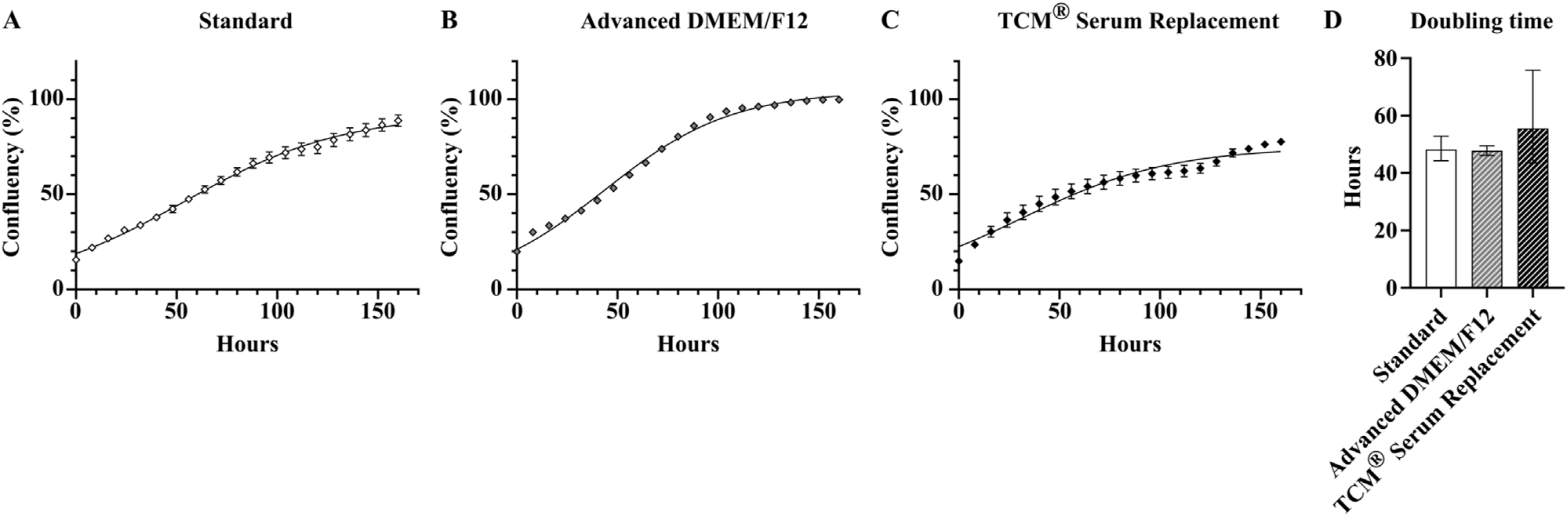
Analysis of growth properties of serum-free cultivated HepG2. Growth curves of cells cultivated either under **(A)** serum containing conditions, **(B)** in Advanced DMEM/F12 (ThermoFisher, middle) or **(C)** DMEM/F12 supplemented with TCM^®^ defined serum replacement (MP Biomedicals, right). **(D)** Doubling times of serum-free cultivated cells and cells cultivated in standard medium calculated from logarithmic phase (8 h–64 h) of growth curves using an exponential (Malthusian) growth equation (R squared Standard: 0.993, R squared Advanced DMEM/F12: 0.998, R squared TCM^®^ Serum Replacement: 0.935). Data are presented as mean ± range. Ordinary one-way ANOVA with Tukey correction for multiple comparison was used to assess statistical significance (N = 1; n = 4).

Based on these specific criteria, of the ten different products used in this experiment, only two were suitable for long-term serum-free culture of HepG2. These two products, Advanced DMEM/F12, ThermoFisher (serum-free medium 1) and TCM^®^ Serum Replacement, MP Biomedicals (serum replacement 2) were able to maintain HepG2 cells with their usual growth characteristics and no significant changes in morphology for more than 25 passages (Figure 3). The average doubling time of cells cultivated in Advanced DMEM/F12 was comparable to cells cultured in FBS containing medium (47.7 ± 1.7 h vs. 48.2 ± 4.6 h, respectively), while the doubling time for cells cultured in TCM^®^ Serum Replacement was slightly higher (55.5 ± 20.2 h) (Figure 4).

Of all other products, two additional products (serum-free medium 2 and serum replacement 4) displayed a limited long-term culturing capacity, with cells surviving without alterations in growth behavior for eleven or eight passages, respectively. All other products were only suitable for short-term culture or not suitable at all. Of these six media, immediate cell death was observed in one condition (serum-free media 4), while death after the first splitting process was observed in another (serum replacement 1). In two conditions (serum-free media 5 and serum-replacement 5) drastic changes in morphology were observed, while in the last two conditions, cell growth was significantly reduced leading to termination of the cultivation of cells in these conditions (see Supplementary Figures S2–S4).

**FIGURE 5:**
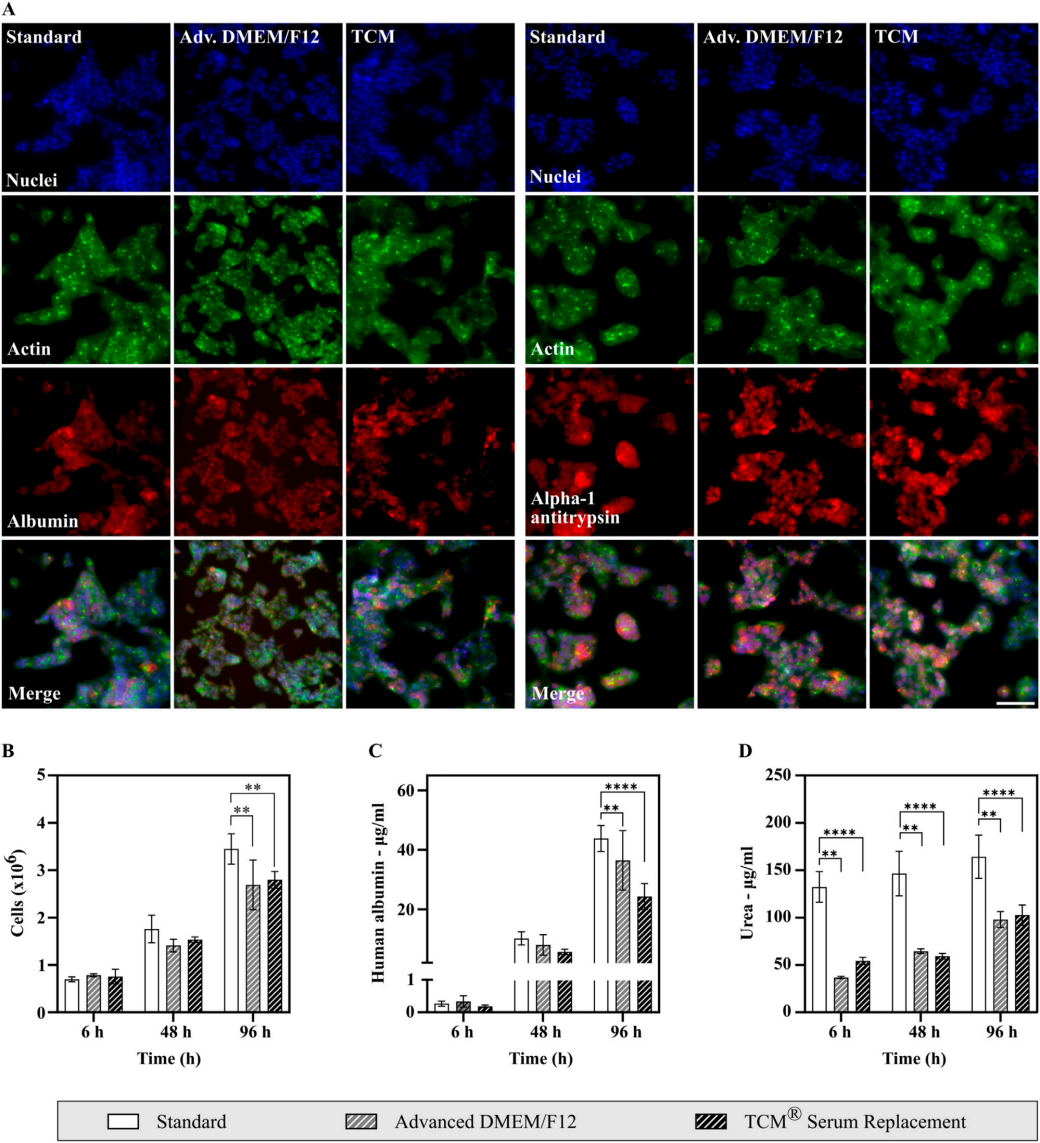
Analysis of HepG2 cell marker expression, albumin production and urea secretion of cells cultivated in either FBS containing medium or serum-free medium. **(A)** Analysis of HepG2 markers using high content imaging. Cells are shown in ×20 magnification. Scale bar: 100 µm. Cells were fixed using paraformaldehyde and subsequently stained with DAPI (nuclei), Actin Phalloidin Alexa 488 (Actin), and either Rabbit ALB (H126) polyclonal antibody (Albumin, left) or Rabbit IgG Alpha-1-Antitrypsin Polyclonal Antibody (Alpha-1 Antitrypsin, right) in combination with Alexa Fluor 680 goat-anti-rabbit as secondary antibody. **(B–D)** Assessment of albumin and urea secretion. Supernatant samples were taken, and cells counted after 6 h, 48 h and 96 h (N = 3, n = 2). Statistical significance was assessed using a two-way ANOVA with Dunnett´s correction for multiple comparison. **(B)** Cell density throughout the experiment. **(C)** Human albumin concentration measured from cell culture supernatants. Albumin production was shown for all conditions. **(D)** Urea secretion measured from cell culture supernatants. Cells in all conditions display urea secretion. Higher concentrations of urea in the standard condition can be explained by high urea concentrations present in fetal bovine serum.

To assess common HepG2 characteristics and functionalities, cells cultured in Advanced DMEM/F12 and TCM^®^ Serum Replacement were stained for two typical HepG2 markers (albumin and alpha 1-antitrypsin) and compared to cells cultivated in FBS containing medium (Hep G2 [HB-8065] | ATCC) (Figure 5A). Both markers were expressed under all conditions, mainly in the cytoplasm as expected.

Additionally, albumin and urea secretion were analyzed from cell culture supernatants (Figures 5B–D). Samples were taken after 6 h, 48 h and 96 h. Human albumin concentrations increased significantly throughout the experiment in all conditions, showing that HepG2 cells produce and secrete albumin. Albumin concentrations were lower in serum-free conditions, however if corrected for cell density, albumin production was highest in Advanced DMEM/F12 (27.11 µg/10^6^ cells after 96 h) with no significant difference to cells cultivated under FBS containing conditions (25.41 µg/10^6^ cells after 96 h, see Supplementary Figure S5). A lower albumin production was only observed for TCM^®^ Serum Replacement (17.39 µg/10^6^ cells after 96 h).

Similarly, increasing urea concentrations were measured in cell culture supernatant samples from all conditions. A significant difference in concentrations was observed between cells cultivated under FBS containing conditions and serum-free conditions. This can be explained by high urea concentrations present in fetal bovine serum, thus increasing the total concentration of urea in cell culture supernatants. Therefore, to compare conditions independently of the corresponding medium fold changes were calculated (Supplementary Figure S5). Fold changes were highest in Advanced DMEM/F12 with a 2.65-fold increase after 96 h. In cell culture supernatants obtained from cells cultured under FBS containing conditions, urea concentrations increased 1.25-fold, while an increase by 1.90-fold was observed in samples of cells cultivated in TCM^®^ Serum Replacement.

As serum-free cultivated cells display HepG2 typical growth characteristics, morphologies, marker expression as well as albumin and urea secretion, cells in both conditions were used to further assess HepG2 cells in common toxicological assays.

### 3.3 Functionality assessment in non-clinical safety assays

**FIGURE 6:**
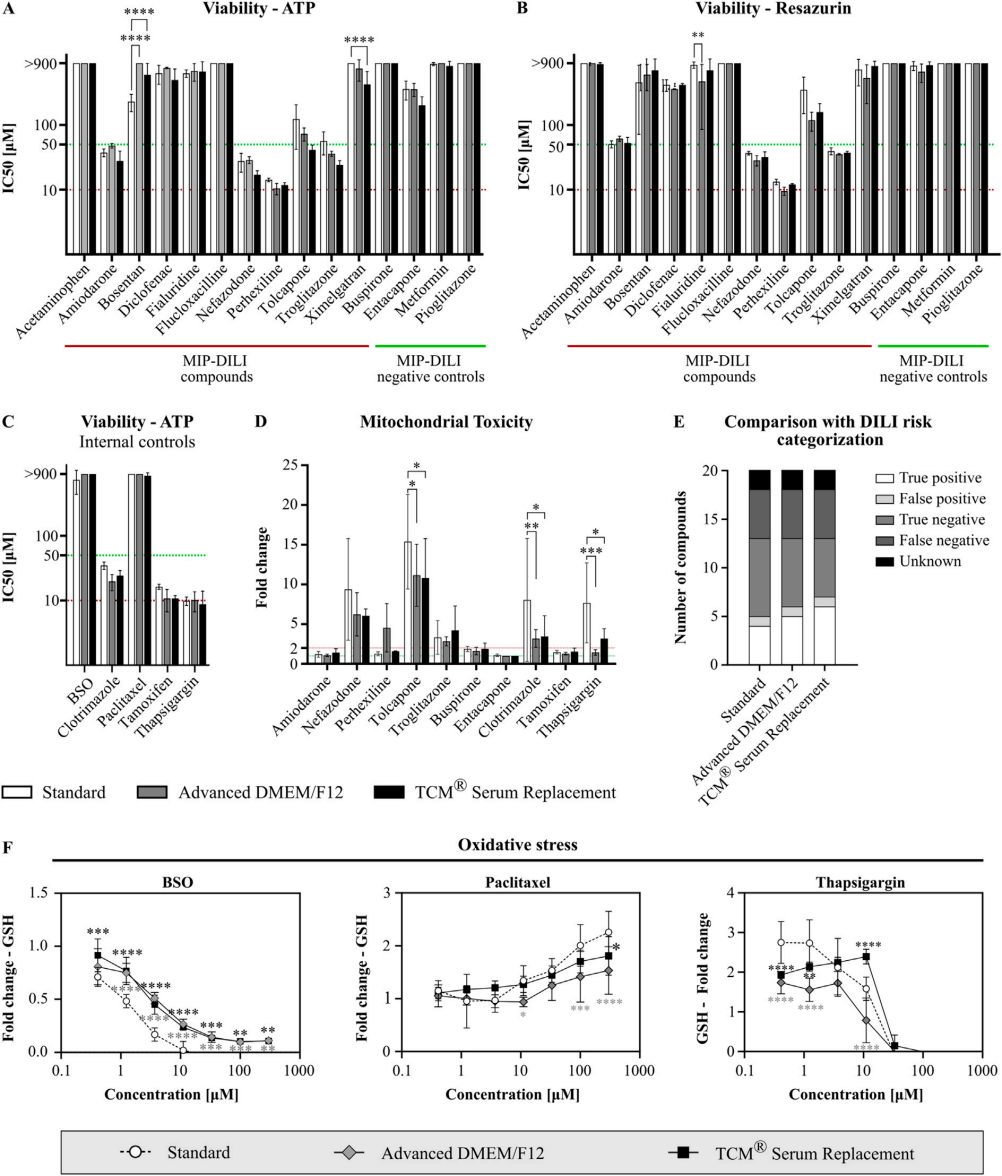
Functionality assessment of serum-free cultivated HepG2. Cells were treated with different reference compounds in a threefold dilution series and subsequently analyzed for viability (N = 3, n = 3), mitochondrial function (N = 3, n = 3), and oxidative stress (N = 2, n = 3). IC_50_ values were obtained from dose response curves, calculated using a log(inhibitor) vs. response model (variable slope, four parameters, bottom constraint = 0). Statistical significance was assessed using a two-way ANOVA with Dunnett´s correction for multiple comparison for all assays. **(A)** Viability assay after treatment of HepG2 cells with MIP-DILI compounds using ATP as read-out (CellTiter Glo Assay). **(B)** Viability assay after treatment with MIP-DILI compounds using the reduction of resazurin to resorufin as read-out (Alamar Blue Assay). **(C)** Viability assay after treatment of HepG2 cells with additional, internal control compounds using ATP as read-out. **(D)** Mitochondrial toxicity assay (Glucose Galactose assay). Cells were treated with either MIP-DILI compounds or internal control compounds. Fold changes between the glucose and galactose condition are used as read-out. Only compounds with a fold change >1 are shown. **(E)** Comparison of *in vitro* data (CellTiter Glo Assay) with FDA categorization of the used reference compounds. Serum-free cultivated cells identified 1 or 2 compounds as a potential DILI risk, which were missed in the FBS containing condition. **(F)** Oxidative stress assay for selected reference compounds (BSO, paclitaxel, and thapsigargin). GSH fold changes are used as read-out. For most compounds, GSH fold changes correlated with viability, indicating no oxidative stress. Viability independent changes in GSH content were only observed for BSO, paclitaxel and thapsigargin. Generally, serum-free cultivated cells displayed a lower oxidative stress response.

Functionality of HepG2 cells cultivated in either Advanced DMEM/F12 or TCM^®^ Serum replacement was assessed using different reference compounds in different drug response assays. MIP-DILI compounds (positive and negative) were selected from Dragovic et al. (2016). Additionally, internal reference compounds were used. All compounds are depicted in Table 2. Two assays focusing on overall cell viability were performed using either ATP (CellTiter Glo Assay) or reduction of resazurin to resorufin (Alamar Blue Assay) as a read out. In the CellTiter Glo Assay, cells were treated with 20 different compounds. A slightly higher sensitivity was observed for the serum-free conditions with 6 compounds (standard containing FBS) vs. 7 compounds (Advanced DMEM/F12) vs. 8 compounds (TCM^®^ Serum replacement) displaying an IC_50_ below 50 μM, the internally set threshold for potential DILI risk (Figures 6A, C). When compared to the human DILI categorization according to the FDA, cells in standard medium identified 4 compounds correctly, missed 8 and identified one negative compound as potentially positive. In the serum-free conditions, one or two compounds were additionally identified as DILI risks, respectively (Figure 6E). Whether the higher sensitivity is due to altered drug response or simply due to less media components binding to the drug, thereby increasing the compound concentration reaching the cells should be addressed in future experiment. For the second assay assessing viability (Alamar Blue Assay), 16 compounds were used. Similar IC_50_ values were obtained for all tested compounds, and 4 compounds were identified with an IC_50_ below 50 µM in all conditions (Figure 6B).

Two further assays were performed to assess potential mitochondrial toxicity and oxidative stress (Figures 6D, F; Supplementary Figure S6). In the Mitochondrial toxicity assay, comparable read-outs were obtained for cells cultivated in FBS containing medium and TCM^®^ serum replacement with 4 shared compounds displaying a fold change higher than 2 throughout all conditions. Generally, fold changes were higher in the standard FBS condition, however especially when comparing standard cells with TCM^®^ Serum Replacement cultivated cells, the same compounds were identified as potential mitochondrial toxins. Differences for two compounds, perhexiline and thapsigargin, were observed for cells cultured in Advanced DMEM/F12. While perhexiline was only identified as a potential mitochondrial toxin in Advanced DMEM/F12 cultivated cells, thapsigargin was below the two-fold threshold, while above in the other conditions. While for perhexiline, mitotoxicity is not reported as a primary mechanism *in vivo*, protonated perhexiline was shown to accumulate in the mitochondrial membrane, resulting in uncoupling of mitochondrial oxidative phosphorylation, inhibition of complexes I and II, and subsequent decreased ATP formation (Dragovic et al., 2016). To further address this, more experiments should be conducted to assess whether the observed differences are caused by an altered drug response or are an effect of temporal resolution.

In the oxidative stress assay, changes in GSH caused by most compounds correlated with a decrease in viability, therefore we selected several reference compounds displaying different responses in the oxidative stress assay (GSH changes correlating with viability, GSH increase independent from viability and GSH decrease independent from viability). (Supplementary Figure S6). From these selected compounds, viability dependent changes in GSH were observed for buspirone, diclofenac, nefazodone and perhexiline and comparable results were obtained for all conditions (Supplementary Figure S6). Additionally, treatment with two compounds induced an increase in GSH either at low concentrations (thapsigargin) or at high concentrations (paclitaxel). For both serum-free media, the observed increase was significantly lower compared to cells cultured under serum-containing conditions (Figure 6F). A strong decrease in GSH, independently from viability, was only observed for buthionine sulfoximine (BSO). Again, changes in GSH content were less profound compared to cells cultivated under serum containing conditions (Figure 6F); therefore, an adaption of assay specific thresholds might be necessary when planning to assess oxidative stress in serum-free conditions.

Overall, cells cultured in serum-free conditions displayed a comparable drug response in *in vitro* toxicology assays assessing viability, mitochondrial toxicity, and oxidative stress response upon compound treatment. Differences observed regarding mitochondrial toxicity and oxidative stress between serum-free cultured cells and cells cultured in FBS containing medium should be addressed in future experiments.

### 3.4 Assessment of intracellular drug response markers

Additionally to non-clinical safety assays, we assessed the effects of test compounds on a specific set of protein markers relevant for cell health. Seven different markers, involved in cell death/apoptosis (PARP), autophagy (LC3B), stress response (HSP70), oxidative stress (HIF-1-alpha), cell division (Histone H3, pSer10), transcription (RNA Pol II, pSer2) and protein synthesis (EIF4B, pSer422) were compared after treatment with either assay specific control substances (bafilomycin, geldanamycin, actinomycin D or deferoxamine) or three previously tested MIP DILI (negative) controls (amiodarone, perhexiline, and metformin).

**FIGURE 7:**
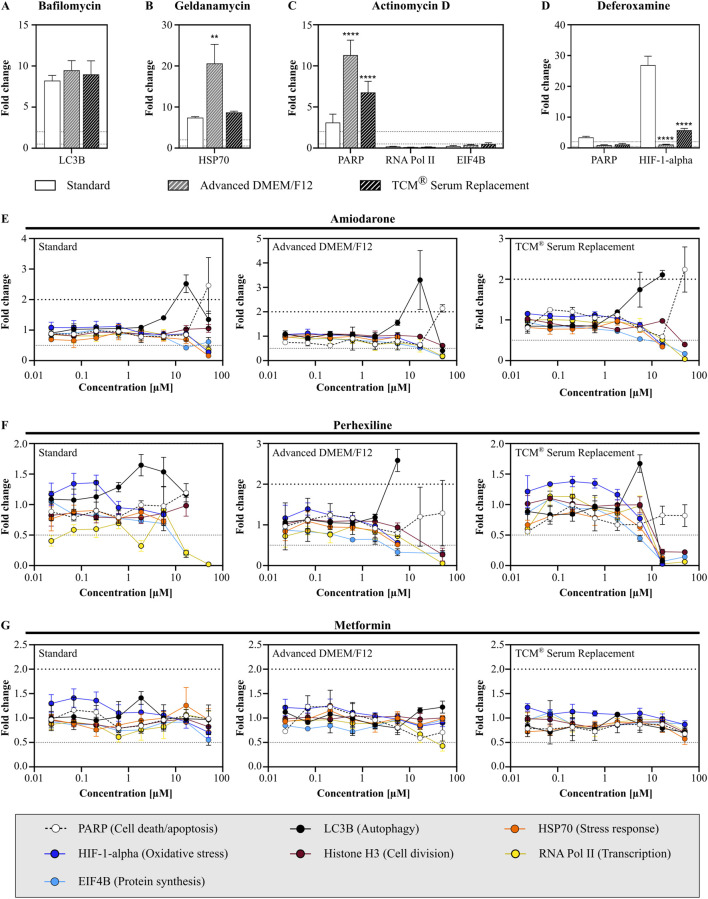
Assessment of intracellular drug response markers after compound treatment. Cells were treated with either assay specific control compounds **(A)** bafilomycin, **(B)** geldanamycin, **(C)** actinomycin D or **(D)** deferoxamine or MIP-DILI (negative) compounds **(E)** amiodarone, **(F)** perhexiline and **(G)** metformin. Protein markers were measured after 24 h and included PARP (cell death/apoptosis), LC3B (autophagy), HSP70 (stress response), HIF-1-alpha (oxidative stress), p-serine of histone H3 (cell division), p-serin 2/5 RNA Polymerase II (transcription), and p-serin 422 EIF4B (protein synthesis). ANOVA with Dunnett correction for multiple testing was used to assess statistical significance (N = 1, n = 3).

When treated with assay specific test substances, cells showed similar response to bafilomycin, with a strong increase in LC3B levels (Figure 7A). After treatment with geldanamycin, cells cultivated in Advanced DMEM/F12 showed a significantly stronger response compared to cells cultivated either in standard medium or TCM (Figure 7B; fold change: 7.4 vs. 20.6 vs. 8.7). Furthermore, cells in serum-free media showed a higher response to actinomycin D (fold change: 3.1 vs. 11.3 vs. 6.6), which was accompanied with a significant decrease in RNA Pol II and EIF4B levels in all conditions (Figure 7C). When treated with deferoxamine, an oxidative stress inducer, only cells in standard medium and TCM showed elevated HIF-1-alpha levels, with standard cells displaying a significantly stronger oxidative stress response, accompanied with increased PARP level, which was not observed in the serum-free media (Figure 7D).

After treatment with the MIP-DILI compounds (amiodarone, perhexiline and metformin), cells displayed a similar drug response across all media. Treatment with amiodarone resulted in elevated PARP levels at the highest concentration, accompanied by a decrease in RNA Pol II, EIF4B, and HSP70 (Figure 7E). A significant decrease in multiple markers indicates that viability was significantly decreased, which given the previously determined IC_50_ values was expected. Furthermore, elevated LC3B levels were observed in the second highest concentration, similar in all conditions. Both, PARP and LC3B levels did not differ significantly between the different conditions.

After treatment with perhexiline, cell death again occurred at concentrations ≥16.7 µM, resulting in multiple parameters either being drastically reduced or below the detection limit, again in accordance with previous results (Figure 7F). A significant increase in PARP was only observed for cells cultivated in Advanced DMEM/F12, with a significant difference to both standard medium and TCM^®^ Serum Replacement. Treatment with Metformin did not result in elevated markers, only a slight decrease in RNA Pol II levels were observed at the highest concentration tested in cells cultivated in Advanced DMEM/F12 (Figure 7G).

Taken together, cells cultivated in Advanced DMEM/F12 and TCM^®^ Serum Replacement showed stronger drug responses when assessing markers related to cell death/apoptosis and stress response, and dependent on the compound a stronger or similar autophagy response compared to cells cultivated in the standard condition. In contrast, the oxidative stress response was significantly lower in both serum-free media. This was already observed in the previously described oxidative stress assay, measuring GSH content, and should be addressed in further experiments. However, oxidative stress was only observed after treatment with deferoxamine, therefore, more oxidative stress inducers should be tested on HepG2 cells with multiple concentrations and time points, to understand whether cells are indeed less sensitive to oxidative stress or if the oxidative stress response is simply shifted in time and may be observed at different time points.

### 3.5 Comparison of freezing media

After confirming functionality of serum-free cultivated cells, another important aspect was to evaluate the possibility of cryo-preservation of cells for later use. Standard freezing media usually contain 10%–20% FBS as well as DMSO, glycerin or other substances as anti-freeze agent (Gstraunthaler and Lindl, 2013). In serum-free cell culture, the protective characteristics of the serum are absent, which is why an evaluation of different freezing media should be performed to ensure high cell recovery and viability after thawing. Therefore, four different serum-free freezing media were tested. Three are commercially available (see Table 3), while the fourth medium was a self-made freezing medium, consisting of 45% conditioned medium, 45% fresh medium and 10% DMSO. After thawing of the cells, cell recovery was determined, and assessment of growth and morphology was performed after 24 h and 72 h, respectively.

**FIGURE 8:**
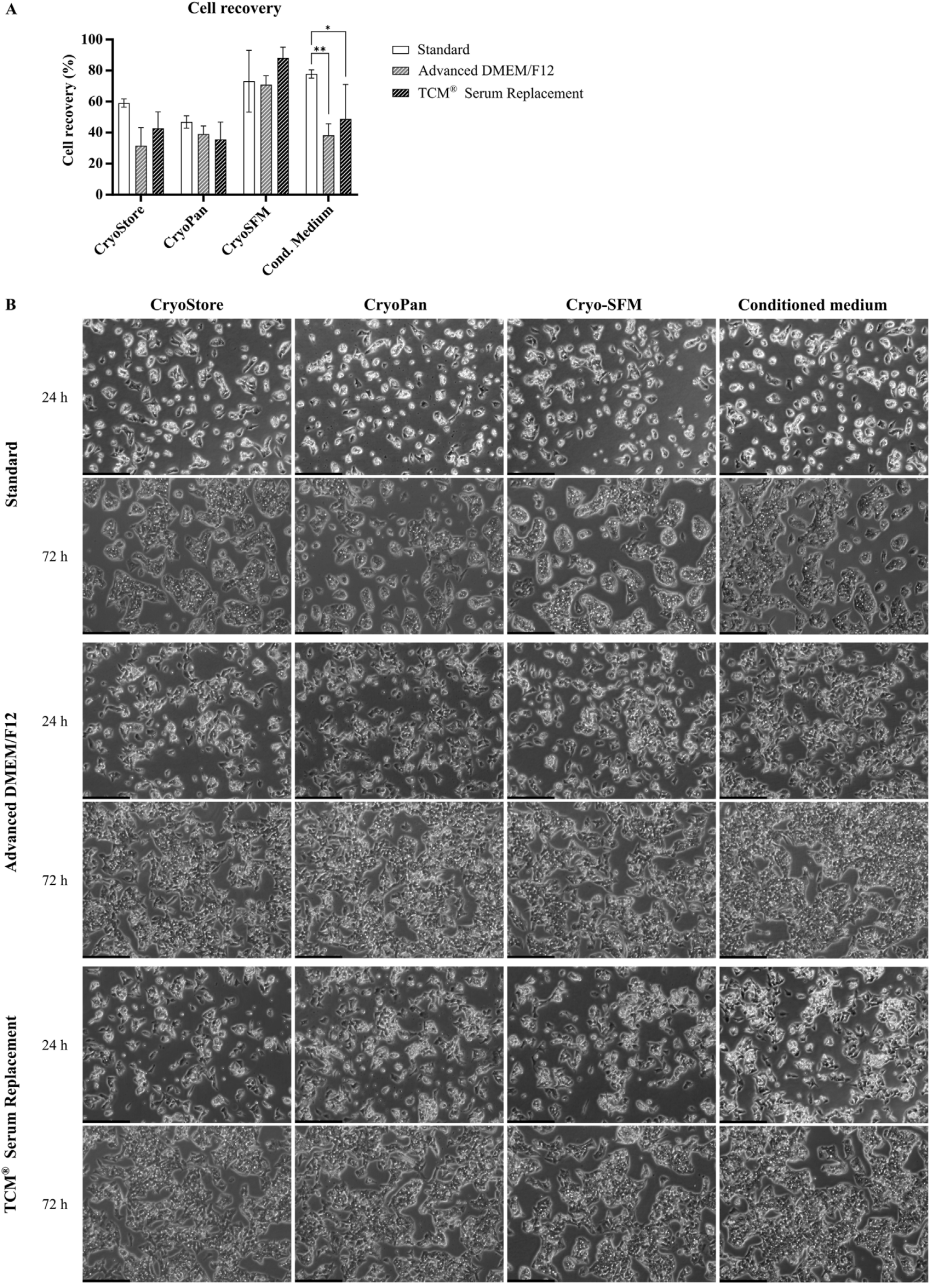
Comparison of freezing media. **(A)** Recovery rate of cryopreserved cells previously cultivated in serum-containing medium (Standard), Advanced DMEM/F12 or TCM^®^ Serum Replacement. Four different freezing reagents, CryoStore, CryoPan, Cryo-SFM or a self-made freezing medium based on conditioned medium, were compared. Cell recovery was highest for Cryo-SFM (across all conditions), and conditioned medium (only Standard and TCM). Two-way ANOVA with Dunnett correction for multiple testing was used to assessed statistical significance. **(B)** Microscopic images of thawed cell taken after 24 h and 72 h. Scale bar: 250 µm.

Cell recovery was highest for Cryo-SFM (77.37% ± 9.37% across all conditions), and conditioned medium (only Standard and TCM^®^ Serum Replacement), while recovery in CryoStore and CryoPan was generally lower (See Figure 8A). Between Standard cells and serum-free cultivated cells, significant differences for recovery were only observed for conditioned medium (Standard: 77.81% ± 2.65%; Advanced DMEM/F12: 38.25% ± 7.43%; TCM Serum Replacement: 48.75% ± 22.27%).

Microscopic images taken after 24 h showed slight differences between serum- and serum-free cultivated cells as already previously observed (Figure 8B). Cells cultured in serum-containing medium showed already small islands, while cells cultivated in serum-free media grew further apart with less cell-cell interactions. No significant differences in cell growth or morphology were observed between the different freezing media, however, particles were observed in all images of cells frozen in CryoPan, which could have originated from the freezing media itself or cell debris.

After 72 h, cells in all condition showed significant cell growth with clear HepG2 typical island formations. Again, islands in serum-free conditions were less dense compared to cells cultivated in serum-containing medium resulting in a higher confluency. Between the different freezing media, no differences were observed.

Taken together, HepG2 cells can easily be cryopreserved using different freezing media resulting in similar growth behavior and similar morphology after thawing. While the recovery rate differed significantly between the different freezing reagents, cell growth after seeding was not impacted.

## 4 Discussion

As the 3R principles are playing an increasing role in research and drug development, and are gaining increasing attention in society and politics, *in vitro* technologies are considered a major replacement tool to avoid/reduce animal experimentation (Jochems et al., 2002; van Zutphen et al., 2001; Weihe, 1985). Nevertheless, animal components are still commonly used in cell culture systems to maintain and proliferate cells, causing both scientific and ethical concerns (Gospodarowicz and Moran, 1976; Hughes et al., 2010; Yao and Asayama, 2017). To fully exploit the potential of *in vitro* technologies as key replacements for animal experimentation, avoiding animal components completely is out of the question. However, since especially FBS is an incredibly complex mixture of nutrients and is until today poorly defined, replacing FBS in cell culture comes with the major task of finding a suitable medium to not only maintain morphology and growth behavior, but cellular functionality (van der Valk et al., 2010).

When deciding to switch out serum containing media with serum-free media, scientists are faced with the question whether designing media themselves or using commercial options. This decision might be influenced by many different aspects and relies heavily on the corresponding situation. Self-made media have the advantage of full control over the formulation (van der Valk et al., 2010). This not only means knowing the full formulation, which is usually not the case for commercial options, but also that media can be easily adapted in case changes are needed, e.g., for a new cell line or in case of shortages in supply (Rafnsdóttir et al., 2023; van der Valk et al., 2010; Weber et al., 2024; Yao and Asayama, 2017). However, designing an animal-component free medium can be highly complex, dependent on the nutritional needs of a certain cell line or cell type as well as assay dependent cellular functions which need to be retained (Cervera et al., 2011; Karnieli et al., 2017; Skrivergaard et al., 2023; van der Valk et al., 2018; Yao and Asayama, 2017). If designing a self-made medium is the preferred option, scientists can significantly benefit from the Fetal Calf Serum free Database (RRID:SCR_018769; https://fcs-free.org), offered by the 3Rs-Centre ULS in collaboration with Animal Free Research United Kingdom.

If setting up a serum-free medium is out of the scope, scientists can rely on commercially available media. In this case, scientists benefit from the usual ready-to-use format, except for few additional supplements, meaning less time and effort needs to be spent (Lindroos et al., 2009; Tan et al., 2015). Furthermore, variability in media composition and costs are significantly decreased due to the tightly regulated and economically effective processes during media production (van der Valk et al., 2018). However, these commercial options have the strong disadvantage of proprietary formulations and the possibility of changes in the formulations without notice (Yao and Asayama, 2017). Furthermore, there is still a significant supply vs. demand problem, with an increasing demand for animal-component free media, while the supply for fully animal-derived component free media however is not sufficient (Brunner et al., 2010). Unfortunately, commercially available media are often designed for one particular cell type or cell line, while media suitable for several cell types are less common.

For this study, designing a media was not the preferred option, therefore different commercially available serum-free media and serum replacements were selected for comparison, using one of the major work horses in early toxicity testing, the HepG2 cell line (Vinken and Rogiers, 2015). During the media selection process, the previously mentioned demand vs. supply chain was highly noticeable with a total number of 5 fully animal-component free or xeno-free products deemed suitable for the planned experiments. Due to the small set of available media, the range was enlarged by including serum-free media as well, if the percentage of animal-derived components within the media was low. Furthermore, since animal components are not restricted to media, TrypLE™, was used to replace the commonly used dissociating agent trypsin and truncated, recombinant vitronectin was used to ensure attachment of cells to the flask under serum-free conditions.

After selecting the inside adaptation protocol as a suitable adaptation strategy by comparing the different protocols described by van der Valk et al., the selected media were compared regarding their long-term culturing capacity. Most media did not support cellular morphology and HepG2 typical growth rates for a prolonged time, with only two media showing long term culturing capacities comparable to the FBS containing condition, indicating missing nutrients in those media that are essential for supporting HepG2 growth. Nevertheless, the used inside adaptation strategy exerted a significant amount of stress on the cells, and therefore cells might not be capable of significantly altering necessary signaling/metabolic pathways based on the new nutritional supply (van der Valk et al., 2010). Therefore, a softer, less challenging long-term adaptation might render more media suitable, however since the goal of this experiment was to find the best media options, this was not further addressed.

Cells adapted to both front-runner media displayed similar growth characteristics, with comparable doubling times and the same epithelial-like morphology, as is typical for HepG2 cells (Arzumanian et al., 2021). Furthermore, the hepatocyte markers albumin and alpha-1-antitrypsin were equally expressed and albumin and urea secretion were shown for all conditions. Since the HepG2 cell line is commonly used to study cell viability, mitochondrial toxicity or oxidative stress upon treatment with new drug candidates, cellular function of these cells cultivated in either one of the two front-runner media was assessed after treatment with either control substances described by the MIP-DILI consortium or internal control compounds (Arzumanian et al., 2021; Dragovic et al., 2016; Vinken and Rogiers, 2015). Throughout the different toxicology assays, serum-free cultivated cells displayed a similar or slightly elevated drug response in the assays focusing on viability/cell death. Comparing our *in vitro* data with the human DILI categorization according to the FDA, cells cultivated in standard medium identified 4 compounds correctly as having a DILI risk, while 8 DILI-positive compounds were not identified. Serum-free cultivated cells identified one or two additional compounds as DILI risk, showing an improvement of the predictive strength of the HepG2 model. The fact that multiple compounds were falsely identified as DILI negative can be explained by the generally lower expression of drug-metabolizing enzymes and transporters in HepG2 cells in comparison to hepatocytes, as well as the absence of immune cells which are known to mediate toxicity of ximelagatram and flucloxacillin (Arzumanian et al., 2021; Berger et al., 2016; Dragovic et al., 2016; Gerets et al., 2012; Sison-Young et al., 2015; Tyakht et al., 2014; Wiśniewski et al., 2016). For this reason, HepG2 cells are suitable as a comparative early screening model and less suitable to investigate metabolism-mediated toxicity (Ren et al., 2018).

A slightly lower sensitivity of cells cultivated under serum-containing conditions might be explained by the presence of many different factors capable of binding the drug in serum, which might prevent higher concentrations of drugs from reaching the cells. FBS contains between 32–70 g/L total protein, the majority made up by serum albumin, an important carrier protein capable of binding lipids, sterols, trace elements or drugs (Francis, 2010; Gstraunthaler and Lindl, 2013; Mishra and Heath, 2021). If using 10% FBS, this corresponds to 3.2–7.0 g/L (0.32%–0.7% w/v) total protein in the final medium and between 2.0 and 3.6 g/L (0.2%–0.36% w/v) BSA. In both serum-free media, the protein quantity is much lower. Advanced DMEM/F12 contains BSA (AlbuMax^®^ II), human transferrin, and recombinant insulin with a total concentration of ∼0.42 g/L (0.042% w/v) and an albumin concentration of 0.4 g/L. For TCM^®^ Serum Replacement, the formulation is proprietary. However, the manufacturer reports heat treated bovine albumin and transferrin as media components with a final total protein concentration less than 0.1%. The lower protein amount therefore might contribute to the observations made in the assays focusing on viability/cytotoxicity. Nevertheless, changes in sensitivity might also be explained by an altered temporal resolution or even changes in expression of drug metabolizing enzymes. To identify key drivers for these observations, additional experiments should therefore be performed, assessing multiple time points or measuring protein expression of key drug metabolizing enzymes.

More significant differences were observed when assessing oxidative stress. In both serum-free media, response to BSO was less profound when measuring GSH content. When measuring HIF-1-alpha expression, no significant increase in HIF-1-alpha levels was observed in treated vs. untreated cells after treatment with deferoxamine for Advanced DMEM/F12 cultured cells, while cells cultivated in TCM^®^ Serum Replacement showed an increase above the threshold, but still significantly lower compared to cell grown in serum containing medium. This might again be explained by different concentrations of media supplements. Advanced DMEM/F12 contains additional ascorbic acid phosphate, glutathione, and transferrin. While both ascorbic acid and glutathione are antioxidants, transferrin prevents formation of reactive oxygen species by binding free iron (Galaris et al., 2019; Jiang et al., 2020; Mandal et al., 2022; Moritz et al., 2020; Njus et al., 2020; Shen et al., 2021). Furthermore, selen and zinc are supplemented in Advanced DMEM/F12. While not having antioxidant activity themselves, both elements are critical for the activity of antioxidative enzymes, like glutathione reductase and superoxide dismutase (Choi et al., 2018; Kang et al., 2020; Olechnowicz et al., 2018; Tinggi, 2008). Since information regarding the FBS formulation are limited, both concentrations of human serum or FBS were used for comparison with Advanced DMEM/F12 (see Table 4).

**TABLE 4:** Comparison of antioxidative agent concentrations in serum supplemented medium and Advanced DMEM/F12.

Supplement	Reference	Concentration		Concentration in DMEM/F12 + 10% serum	Concentration in advanced DMEM/F12
Ascorbic acid	Human serum	50–60 µM (Khaw and Woodhouse, 1995)	8.8 mg/L	0.88 mg/L	2.5 mg/L
Glutathione	Human serum	2.1 ± 0.0009 µM (Morrison et al., 1999)	0.645 mg/L	0.064 mg/L	1 mg/L
Transferrin	FBS	∼22.5–27.5 µM	1.8–2.2 g/L (Kakuta et al., 1997)	0.18 g/L	7.5 mg/L
Selen	FBS	177.31–481.26 nM	0.014–0.038 mg/L (Gstraunthaler and Lindl, 2013)	1.4–3.8 μg/L	0.005 mg/L (sodium selenite)
Zinc	FBS	0.25 ± 0.02 nM (López-Solís et al., 2022)	16.345 ng/L	0.432 mg/L (zinc sulfate)	0.864 mg/L (zinc sulfate)

Except for transferrin, all other mentioned supplements are present in Advanced DMEM/F12 in higher concentrations compared to DMEM/F12 supplemented with 10% FBS. Therefore, oxidative stress response might indeed be affected. However, to find the definite cause, further experiments are necessary.

Lastly, we want to address the remaining animal-derived components present in both front-runner media. As animal-derived components still compromise the potential of *in vitro* cell culture models to function as replacement tools for animal experimentation, these compounds should be considered as critical even if present in low concentrations (Messmer et al., 2022; Post et al., 2020). While serum-free media are a better alternative for serum-containing media, any remaining animal-derived components should be removed as well in order to be more ethically acceptable. Additionally, removing animal-derived components is preferential also for scientific reasons, as for instance bovine transferrin was shown to contain a xenoantigen (Neu5Gc) contamination which might impact assay outcomes (Heiskanen et al., 2007). Therefore, next steps should include building a self-made medium based on the formulation on Advanced DMEM/F12 which is publicly available. Advanced DMEM/F12 contains only 10 different supplements, with only albumin as an animal-derived component. Bovine serum albumin is commonly used, due to its lower cost (Keenan et al., 1995). However, studies on functionality of human serum albumin or recombinant human albumin in cell culture already showed that HSA is a suitable alternative (Barbaro et al., 2008; Fanali et al., 2012; Francis, 2010; Keenan et al., 1997; Ng et al., 2008). To find a suitable product, it may be necessary to compare different manufacturers due to different ligand-interactions and in case of recombinant albumin potential alterations in post-translational modifications, dependent on the expression host (Mishra and Heath, 2021). A fully animal-derived component-free medium formulation similar, albeit more complex compared to Advanced DMEM/F12, was already published by Rafnsdóttir et al. and supplements used for this medium should be tried first when comparing different media supplements (Rafnsdóttir et al., 2023).

Additionally, as one important factor necessary for replacing animal-derived components in cell culture are manufacturers, users of cell culture media should communicate the needs and demands in animal-derived component free media (van der Valk et al., 2018). Furthermore, convincing scientists to switch to animal-component free media by publishing and presenting solid data showing comparability of results and advertising the advantages of serum-free culture is key to further drive ambitions to replace animal components in cell culture (Duarte et al., 2023).

Taken together, the obtained data strongly support the use of serum-free media when working with HepG2 cells. Generally, growth characteristics and morphology were maintained, while assay data were comparable with those obtained from cells cultivated under standard, FBS containing conditions.

## Data Availability

The raw data supporting the conclusions of this article will be made available by the authors, without undue reservation.

## Supplementary material

SUPPLEMENTARY FIGURE S1 Selection of the front-runner media for serum-free culture of HepG2. Long-term culturing capacity of different serum-free media using the inside adaptation procedure (left) and categorization of serum-free media with the reason of termination if a medium was not classified as suitable for long-term culture.

SUPPLEMENTARY FIGURE S2 Microscopic images of HepG2 cells cultured in different serum-free media or media containing serum replacements on day 3 (serum-free passage 1). Marked conditions were terminated. Scale bar: 100 µm.

SUPPLEMENTARY FIGURE S3 Microscopic images of HepG2 cells cultured in different serum-free media or media containing serum replacements on day 7 (serum-free passage 2). Marked conditions were terminated. Scale bar: 100 µm.

SUPPLEMENTARY FIGURE S4 Microscopic images of HepG2 cells cultured in different serum-free media or media containing serum replacements on day 11/12 (serum-free passage 3). Marked conditions were terminated. Scale bar: 100 µm.

SUPPLEMENTARY FIGURE S5 Analysis of albumin production and urea secretion. Supernatant samples were taken after 6 h, 48 h and 96 h (N = 3, n = 2). Albumin concentrations were normalized to cell count to compare conditions. Urea production was analyzed by calculating the fold change based on samples taken after 6 h in culture. Statistical significance was assessed using a two-way ANOVA with Dunnett´s correction for multiple comparison.

SUPPLEMENTARY FIGURE S6 Oxidative stress assay using the GSH fold change as read-out. Viability data are shown for comparison. Different responses to compound treatment were observed: viability dependent GSH changes, viability independent GSH increase at either high or low compound concentrations or a viability independent GSH decrease.
