# Supplementary figures and images for: Increasing sustainability and reproducibility of in vitro toxicology applications: serum-free cultivation of HepG2 cells
Source: Front Toxicol. 2024 Nov 22;6:1439031. doi: 10.3389/ftox.2024.1439031 (PMC11621109; doi:10.3389/ftox.2024.1439031)

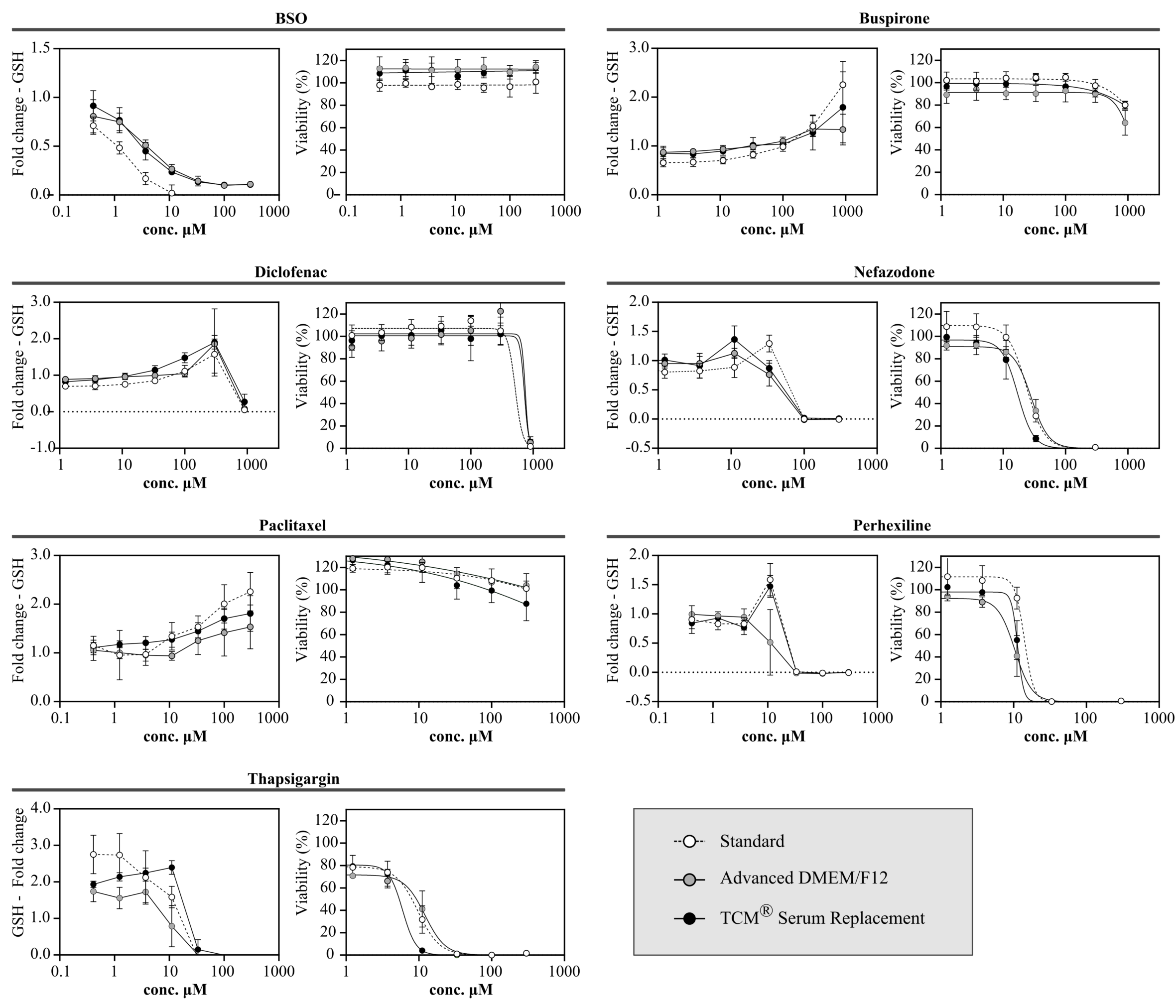

Supplement: Supplementary file 4 [file Image6.tif]

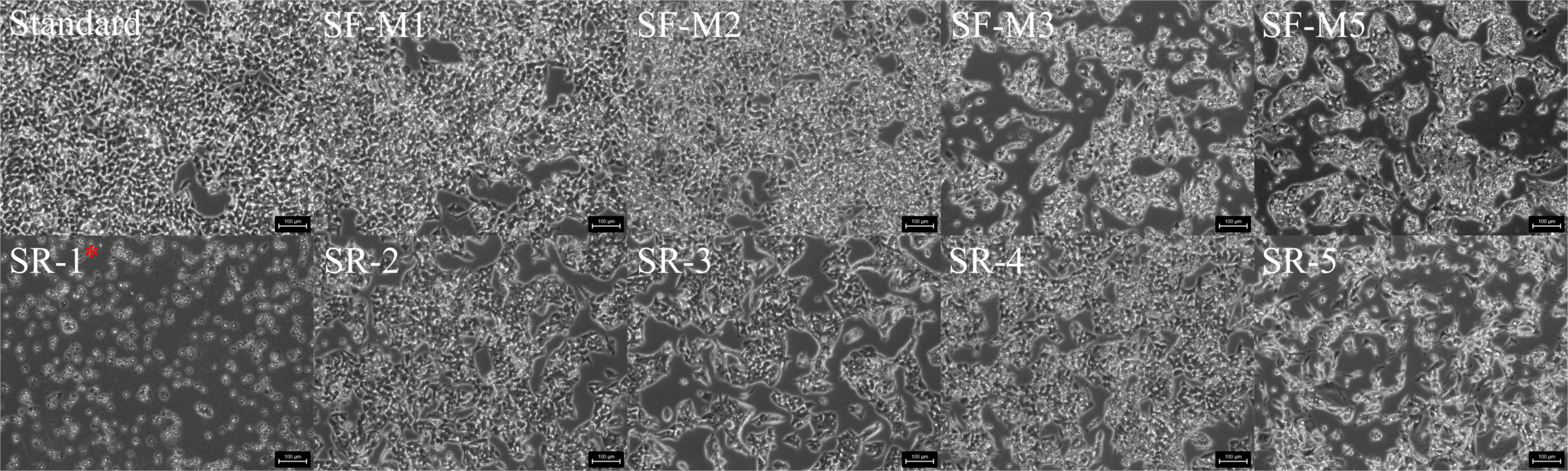

Supplement: Supplementary file 5 [file Image3.tif]

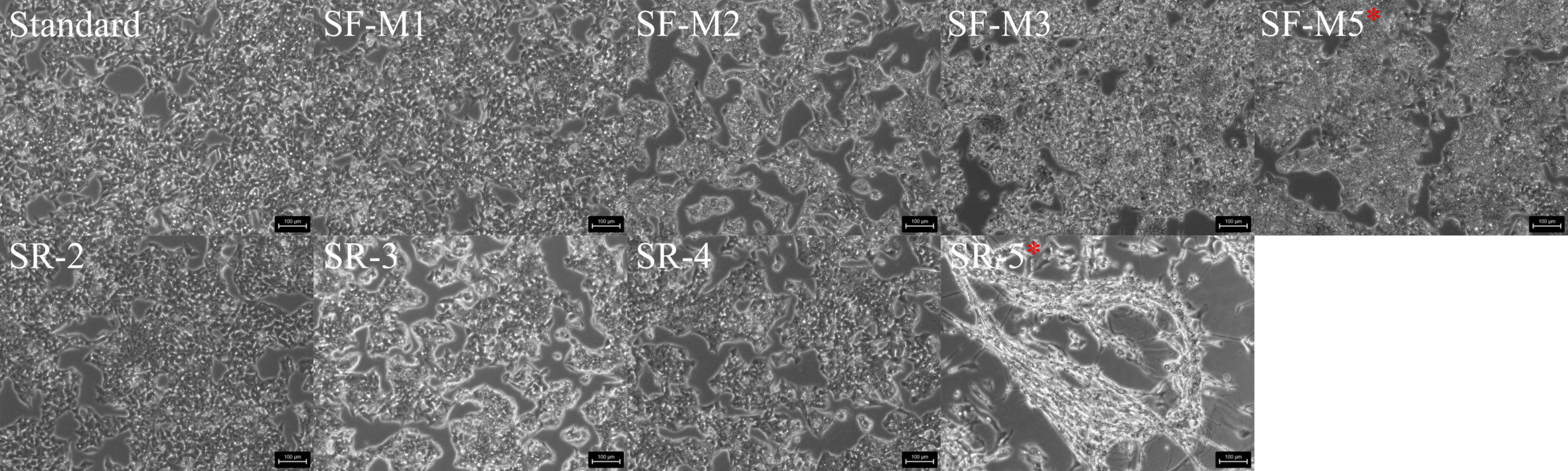

Supplement: Supplementary file 6 [file Image4.tif]

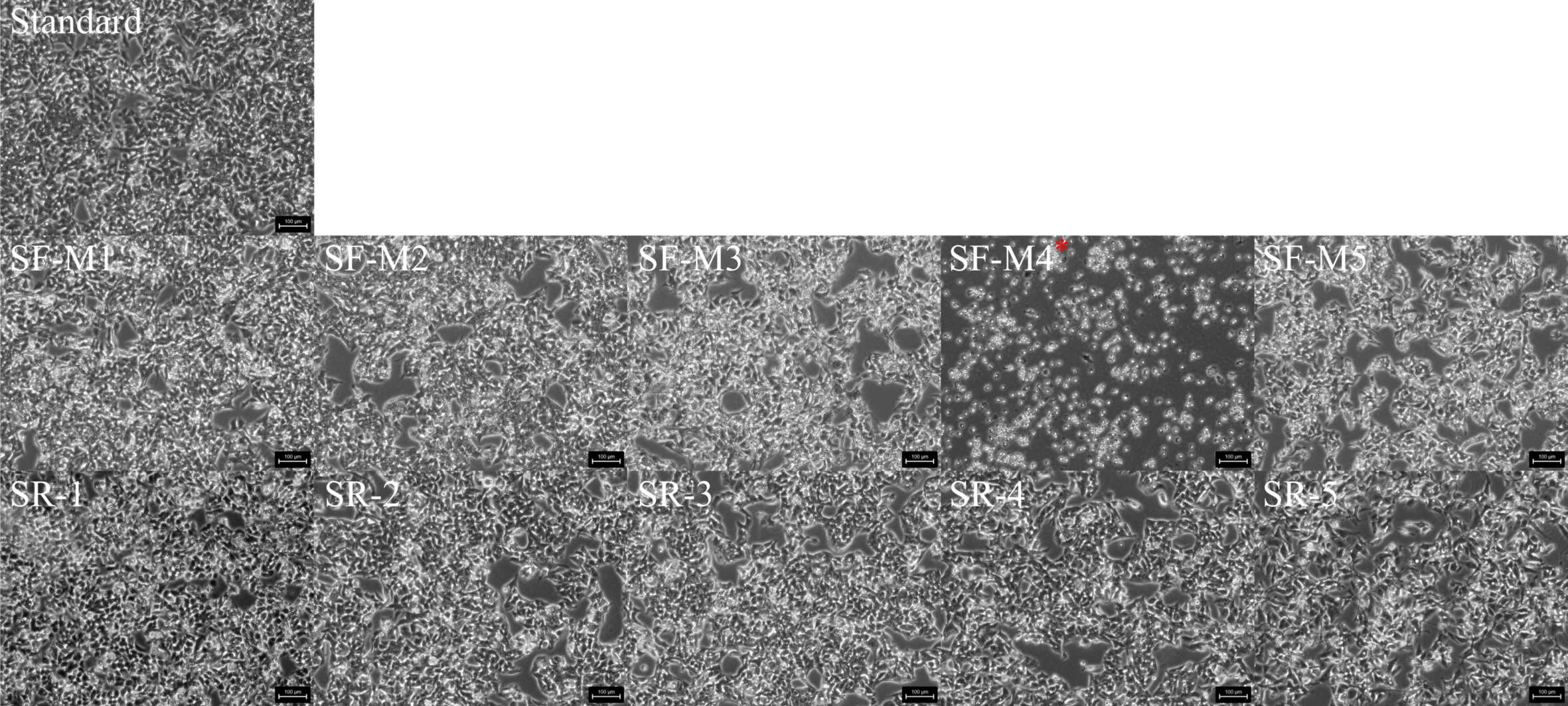

Supplement: Supplementary file 7 [file Image2.tif]

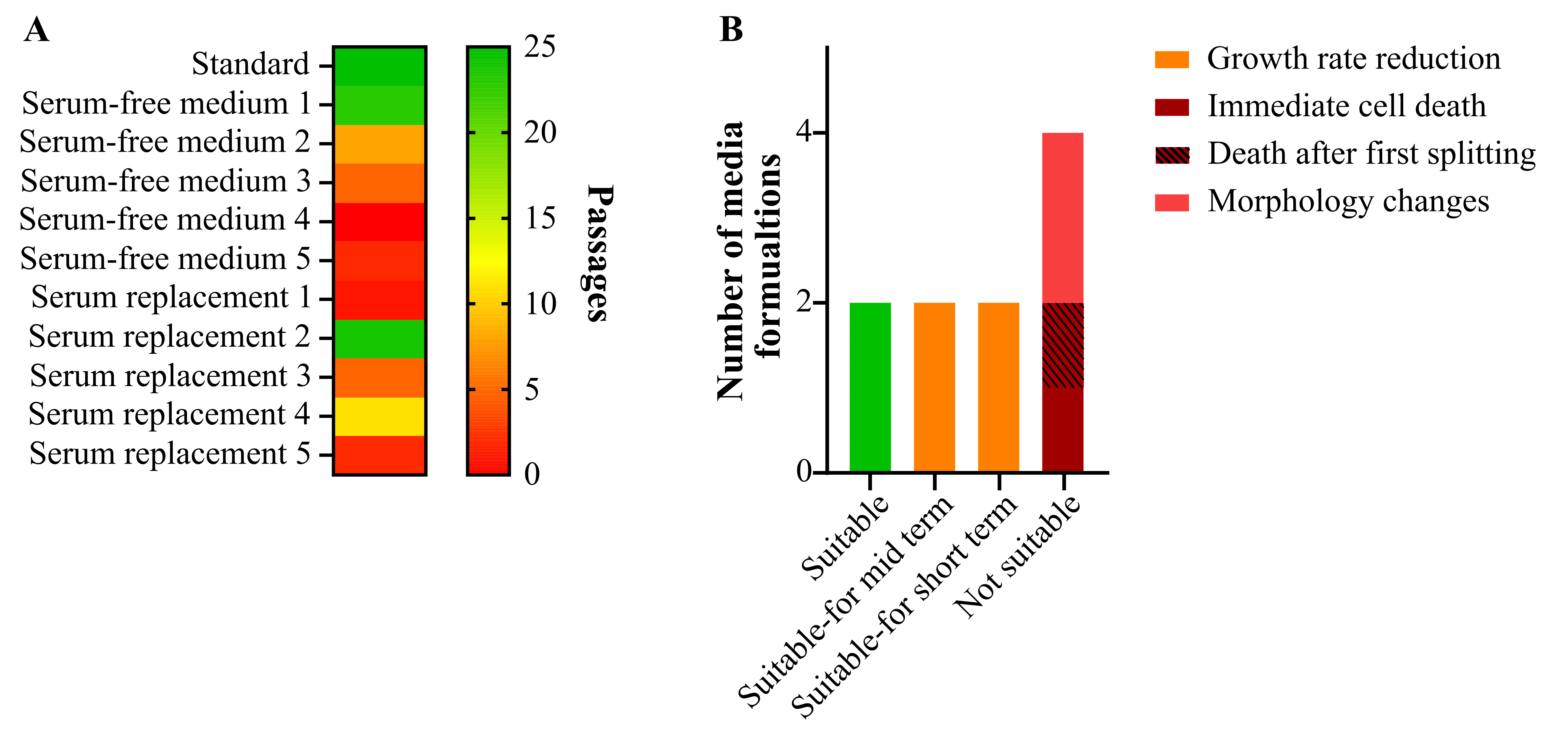

Supplement: Supplementary file 8 [file Image1.tif]

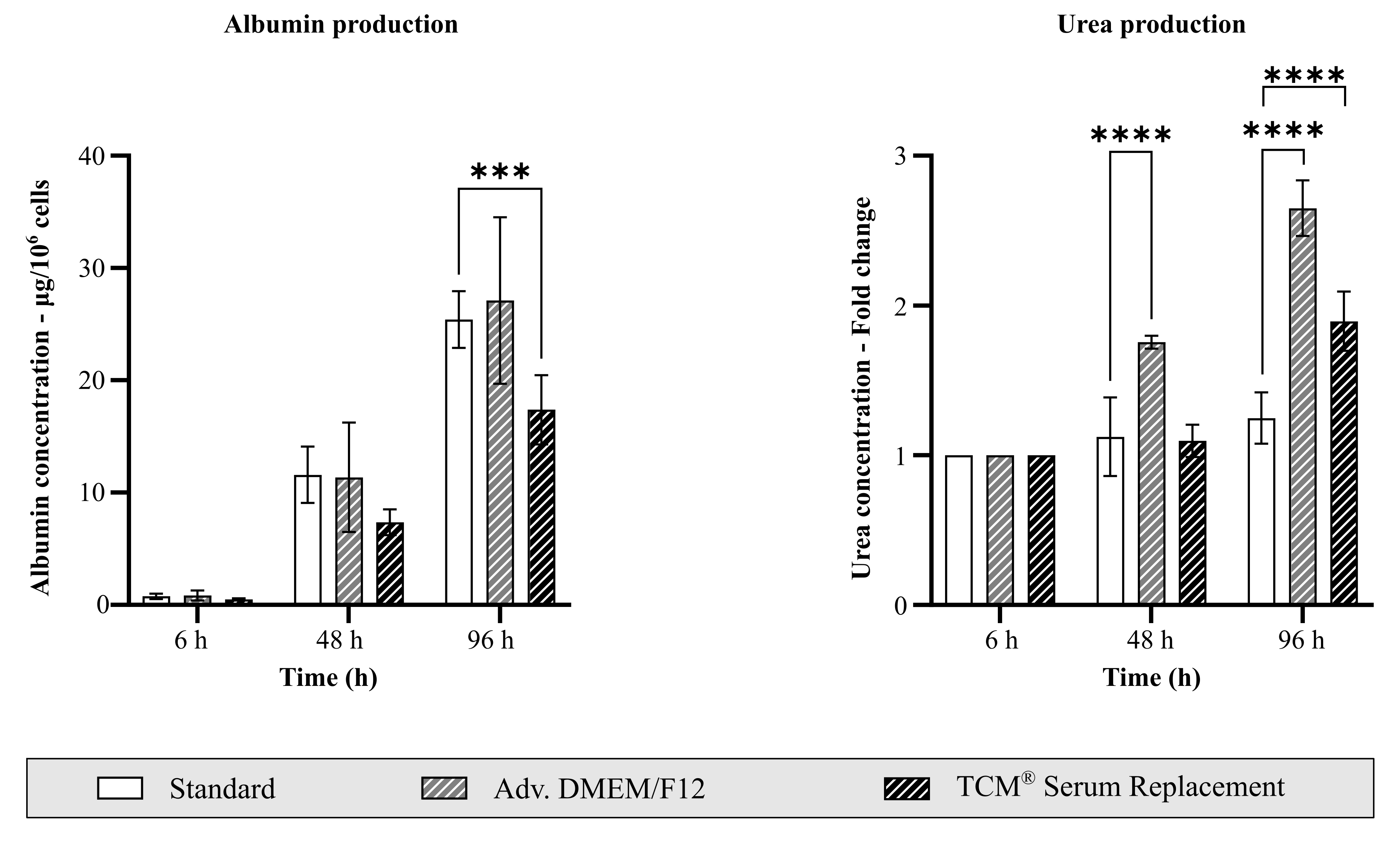

Supplement: Supplementary file 11 [file Image5.tif]
