# Supplementary material for: Increasing sustainability and reproducibility of in vitro toxicology applications: serum-free cultivation of HepG2 cells
Source: Front Toxicol. 2024 Nov 22;6:1439031. doi: 10.3389/ftox.2024.1439031 (PMC11621109; doi:10.3389/ftox.2024.1439031)
Supplement: Supplementary file 10 [file Table4.docx]

Supplementary Table 5. Statistical information on analysis used, including number of technical and biological replicates and statistical tests performed. Biological replicates were done by using cells at different passages.

| **Endpoint/Assay** | **Figure** | **Number of replicates (n = technical, N = biological)** | **Statistical test** | **Post-hoc** |
| --- | --- | --- | --- | --- |
| Comparison of adaptation procedures | Figure 2 A and B | N = Not applicable, as experiment was performed across multiple passages; n = 1 |  |  |
| Growth curves | Figure 4A-C | N = 1; n=4 | - | - |
| Doubling time | Figure 4D | N = 1; n=4 | ANOVA | **Tukey** |
| Cell numbers, Albumin and Urea production | Figure 5C-D | N=3, n=2 | Ordinary two-way ANOVA | **Dunnett** multiple comparisons (all serum-free conditions compared to standard condition) |
| Viability – ATP | Figure 6A and C | N = 3, n= 3 | Ordinary two-way ANOVA | **Dunnett** multiple comparisons (all serum-free conditions compared to standard condition) |
| Viability – Resazurin | Figure 6B | N = 3, n= 3 | Ordinary two-way ANOVA | **Dunnett** multiple comparisons (all serum-free conditions compared to standard condition) |
| Mitochondrial toxicity | Figure 6D | N = 3, n= 3 | Ordinary two-way ANOVA | **Dunnett** multiple comparisons (all serum-free conditions compared to standard condition) |
| Oxidative stress | Figure 6F | N = 2, n= 3 | Ordinary two-way ANOVA | **Dunnett** multiple comparisons (all serum-free conditions compared to standard condition) |
| Measurement of intracellular drug response marker | Figure 7A and B | N=1, n=3 | ANOVA | **Dunnett** |
| Measurement of intracellular drug response marker | Figure 7C-G | N=1, n=3 | Ordinary two-way ANOVA | **Dunnett** multiple comparisons (all serum-free conditions compared to standard condition) |
| Comparison of freezing media | Figure 8A | N=2, n=1 | Ordinary two-way ANOVA | **Dunnett** multiple comparisons (all serum-free conditions compared to standard condition) |
